# Predictive Value of the Pulmonary Artery Pulsatility Index in Pulmonary Arterial Hypertension: REVEAL Analysis

**DOI:** 10.14740/cr2225

**Published:** 2026-06-05

**Authors:** Nicole F. Ruopp, Harrison W. Farber, Mohammad Rahman, Navin K. Kapur, Zeenat Safdar

**Affiliations:** a Division of Pulmonary, Sleep and Critical Care Medicine, Tufts Medical Center, Boston, MA, USA; b Johnson & Johnson, Titusville, NJ, USA; c Houston Methodist Lung Center, Houston Methodist Hospital, Weill Cornell College of Medicine, Houston, TX, USA

**Keywords:** Pulmonary arterial hypertension, Pulmonary artery pulsatility index, Risk assessment

## Abstract

**Background:**

There is growing interest in developing risk assessment tools/metrics to improve treatment, management, and outcomes for pulmonary arterial hypertension (PAH). This study investigated the association of the pulmonary artery pulsatility index (PAPi) with patient characteristics, hospitalization, and mortality.

**Methods:**

Data collected from the US-based Registry to Evaluate Early and Long-Term PAH Disease Management were stratified/analyzed according to baseline PAPi quartiles (36-month follow-up).

**Results:**

In total, 2,711 patients were included. Baseline demographic/clinical characteristics were similar; however, the lowest quartiles (< 3.55; ≥ 3.55 to < 5.5) had higher New York Heart Association/World Health Organization functional class, while those in the highest quartiles (≥ 5.5 to < 9.0; ≥ 9.0) had longer 6-min walk distance. Of 2,414 patients assessed for hospitalization, 1,326 (54.9%) were hospitalized. Lower PAPi correlated with increasing hospitalization probability (≥ 9.0 quartile, n = 291 (48.0%); ≥ 5.5 to < 9.0 quartile, n = 333 (54.8%); ≥ 3.55 to < 5.5 quartile, n = 340 (57.2%); and < 3.55 quartile, n = 362 (59.7%)). Of 681 (28.1%) patients who died, 150 (24.1%) were in the ≥ 9.0 quartile, 154 (25.5%) the ≥ 5.5 to < 9.0 quartile, 157 (26.7%) the ≥ 3.55 to < 5.5 quartile, and 220 (35.9%) the < 3.55 quartile (P < 0.001). Compared with the < 3.55 quartile, there was a 35.6%, 25.8%, and 23.3% reduction in mortality in the ≥ 9.0, ≥ 5.5 to < 9.0, and ≥ 3.55 to < 5.5 quartiles, respectively.

**Conclusions:**

PAPi may be a useful prognostic tool and long-term predictor of clinical events in PAH.

## Introduction

Pulmonary arterial hypertension (PAH) is a progressive disorder associated with elevated pulmonary vascular resistance, leading to right ventricular (RV) failure and death [1]. Although mortality remains high, there has been progress in new treatment strategies [2]. The European Society of Cardiology/European Respiratory Society (ESC/ERS) guidelines recommend that patients are assessed regularly to determine risk of dying, and that treatment is tailored to maintain low-risk status [2].

Attention has become focused on development of multiparametric risk assessment tools. The ESC/ERS three-strata and four-strata risk assessment tools have been validated using data from disease registries [3–7], and other tools have been developed from the US-based Registry to Evaluate Early and Long-Term PAH Disease Management (REVEAL), including the REVEAL 2.0 Risk Calculator and its abridged form, REVEAL Lite 2 [8, 9]. The REVEAL 2.0 score incorporates demographic, laboratory, hemodynamic, and echocardiographic variables, while the REVEAL Lite 2 risk calculator only relies on six noninvasive variables, including functional class, heart rate, systolic blood pressure, 6-min walk distance (6MWD), B-type natriuretic peptide (BNP)/N-terminal proBNP, and renal function. There is growing interest in developing other tools and metrics for routine clinical use, as consistent and accurate evaluation of risk could improve patient outcomes. Future tools could be utilized to inform continuous re-evaluation and re-adjustment of treatment strategies to remain tailored to individual patients [10]. Future tools might also help to identify patients with subclinical disease progression, whose condition can be mistakenly categorized as stable when symptoms indicative of worsening PAH are absent or not identified by current risk assessment tools [11].

RV-pulmonary arterial (PA) coupling is highly prognostic for survival in PAH [12, 13]. RV-PA coupling, assessed by the tricuspid annular plane systolic excursion/systolic pulmonary artery pressure ratio by echocardiography, was added to the 2022 ESC/ERS guidelines, as poor coupling can detect development of RV failure [14]. Another metric, the pulmonary artery pulsatility index (PAPi), is a hemodynamic index defined as PA pulse pressure (i.e., PA systolic pressure minus PA diastolic pressure) divided by right atrial pressure (RAP); it shows the RV adaptive response to increased afterload (RV-PA coupling). Although PA pulse pressure is an invasive measurement [15], PAPi has been proposed as an improved marker for RV-PA coupling [13] and was shown to predict RV failure in patients with acute inferior wall myocardial infarction and left ventricular assist device implantation [16–18]. PAPi has also been associated with mortality and cardiac events in patients with cardiovascular disease [19].

PAPi is widely utilized in cardiac critical care and plays a key role in clinical decision-making [20]; however, most supporting literature remains limited to small, single-center studies [13, 21]. Recent studies show an association between low PAPi values and higher mortality in patients with PAH [13, 22, 23]. The present study investigated the association of PAPi quartiles with baseline demographic and clinical characteristics, hospitalization, and mortality in a large population of patients with PAH from REVEAL (NCT00370214).

## Materials and Methods

### REVEAL study population

REVEAL is an observational registry that enrolled patients with PAH at 55 centers in the United States between March 2006 and December 2009 [24]. Patients were followed for at least 5 years [24]. They had hemodynamically confirmed (per the timing of this analysis) PAH via right heart catheterization (RHC), with mean pulmonary artery pressure > 25 mm Hg at rest or > 30 mm Hg with exercise, mean pulmonary capillary wedge pressure or left ventricular end-diastolic pressure ≤ 18 mm Hg at rest, and pulmonary vascular resistance ≥ 3 Wood units [24]. REVEAL (NCT00370214) was conducted in accordance with the amended Declaration of Helsinki. The REVEAL protocol was reviewed and approved by the Institutional Review Board of each participating center with written informed consent obtained from all patients.

Patients were included in the present analysis if they were ≥ 18 years of age and had at least one RHC ≥ 60 days before enrollment or within 60 days of follow-up. Patients were excluded if they had pulmonary artery wedge pressure > 15 mm Hg or met inclusion criteria during exercise only.

### Study endpoints and statistical analysis

The analysis included patient demographic and clinical characteristics collected at registry entry (baseline). PAPi values were calculated from RHC data at baseline. Patients were stratified according to PAPi quartile (< 3.55, ≥ 3.55 to < 5.5, ≥ 5.5 to < 9.0, or ≥ 9.0). The quartiles were identified using a combination of data visualization techniques and a standard statistical process to examine the distribution and determine the appropriate cut-points. The quartiles were derived from the full analytic sample that included all patients who met the eligibility criteria. An additional analysis using absolute PAPi cut-off values (< 3.55 vs. ≥ 3.55) was included in accordance with similar analyses to date [20].

Group comparisons between PAPi strata for the association of PAPi with baseline demographics, all-cause hospitalization, and all-cause mortality were performed using Pearson’s Chi-squared test. Hospitalization and mortality throughout follow-up were additionally evaluated using Kaplan–Meier analysis; hazard ratios, 95% confidence intervals, and P values were calculated for the lowest PAPi quartile (< 3.55) versus each of the higher three quartiles, or for < 3.55 versus ≥ 3.55 (absolute cut-off values). Hospitalization and mortality were additionally analyzed in a subset of patients with available hospitalization records according to whether they had incident or prevalent PAH: incident cases were defined as patients who received a diagnosis of PAH confirmed by RHC during recruitment to REVEAL, while prevalent cases were defined as patients diagnosed prior to the start of REVEAL.

Continuous variables were summarized using the number of patients, number of missing values, mean, median, standard deviation, range, and first and third quartiles. Categorical variables were summarized using number of patients, number of missing values, frequency counts, and percentages. Calculations of percentages were based on the number of patients with available values, i.e., patients with missing or unknown values were excluded.

To determine the optimal cut-off value for PAPi that best separates mortality of two groups, maximally selected log-rank statistics were employed using the maxstat R package [25]. Kaplan–Meier curves of mortality for the two PAPi groups were generated, and significance testing for between-group differences was performed.

## Results

### Patients

**Figure 1:**
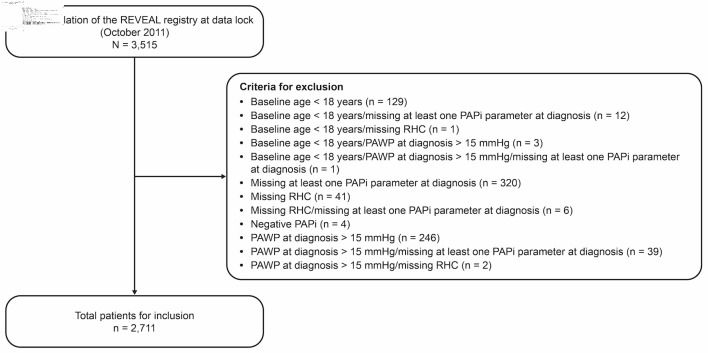
Reasons for exclusion from the study. PAPi: pulmonary artery pulsatility index; PAWP: pulmonary artery wedge pressure; REVEAL: Registry to Evaluate Early and Long-Term PAH Disease Management; RHC: right heart catheterization.

The total population of the REVEAL registry was 3,515 at data lock in October 2011 [24]; from this population, 2,711 patients were eligible for the present analysis. Reasons for exclusion are shown in Figure 1; of note, four patients with negative PA pulse pressures were excluded from the analysis.

### Baseline characteristics by PAPi quartile

There was no substantial difference between PAPi quartiles in any baseline characteristics except New York Heart Association/World Health Organization (NYHA/WHO) functional class; patients in the lowest quartiles (< 3.55 and ≥ 3.55 to < 5.5) generally had more severe functional impairment (Tables 1, 2) [24]. This was also observed for the analysis according to absolute PAPi cut-off value of < 3.55 versus ≥ 3.55 (Supplementary Material 1, cr.elmerpub.com); patients with PAPi values < 3.55 also had higher body mass index and were more likely to be male compared with those with values ≥ 3.55 (Supplementary Material 1, cr.elmerpub.com).

**Table 1:** Baseline Demographic Characteristics by PAPi Quartile

Characteristic	PAPi quartile				
	Overall (N = 2,711)	< 3.55 (n = 678)	≥ 3.55 to < 5.5 (n = 668)	≥ 5.50 to < 9.0 (n = 682)	≥ 9.0 (n = 683)
Age, years					
N	2,711	678	668	682	683
Mean (SD)	52.4 (14.7)	51.4 (13.9)	53.5 (14.4)	53.1 (14.9)	51.8 (15.5)
Median (IQR)	53.0 (42.4–63.3)	51.4 (41.6–61.3)	54.1 (44.0–64.1)	53.5 (42.9–64.3)	52.4 (41.2–63.2)
Age at diagnosis, years					
N	2,711	678	668	682	683
Mean (SD)	49.7 (15.5)	49.1 (14.2)	51.0 (15.1)	50.2 (15.9)	48.6 (16.7)
Median (IQR)	50.2 (39.0–61.1)	49.4 (38.7–59.2)	51.2 (41.2–62.0)	50.5 (40.1–62.4)	49.7 (36.6–60.8)
Sex, n (%)					
Male	561 (20.7)	164 (24.2)	138 (20.7)	132 (19.4)	127 (18.6)
Female	2,150 (79.3)	514 (75.8)	530 (79.3)	550 (80.6)	556 (81.4)
Race, n (%)					
White	1,969 (72.6)	480 (70.8)	489 (73.2)	518 (76.0)	482 (70.6)
Black	347 (12.8)	112 (16.5)	89 (13.3)	72 (10.6)	74 (10.8)
Hispanic	236 (8.7)	48 (7.1)	50 (7.5)	60 (8.8)	78 (11.4)
Asian	86 (3.2)	17 (2.5)	23 (3.4)	13 (1.9)	33 (4.8)
Other	73 (2.7)	21 (3.1)	17 (2.5)	19 (2.8)	16 (2.3)
BMI, kg/m^2^					
N	2,587	640	637	651	659
Mean (SD)	28.3 (7.0)	29.3 (7.4)	29.3 (7.4)	27.9 (6.6)	26.5 (6.1)
Median (IQR)	27.1 (23.2–31.8)	28.4 (24.2–33.1)	28.1 (24.1–33.2)	26.8 (23.2–31.1)	25.7 (21.8–30.1)
Missing, n	124	38	31	31	24
PAH diagnosis, n (%)^a^					
Incident	900 (33.2)	248 (36.6)	243 (36.4)	207 (30.4)	202 (29.6)
Prevalent	1,811 (66.8)	430 (63.4)	425 (63.6)	475 (69.6)	481 (70.4)
Diagnostic status, n (%)^b^					
Newly diagnosed	737 (27.2)	206 (30.4)	199 (29.8)	165 (24.2)	167 (24.5)
Previously diagnosed	1,974 (72.8)	472 (69.6)	469 (70.2)	517 (75.8)	516 (75.5)
NYHA/WHO FC, n (%)					
I	172 (7.1)	26 (4.3)	39 (6.3)	54 (8.9)	53 (8.7)
II	843 (34.6)	181 (29.9)	200 (32.5)	229 (37.9)	233 (38.2)
III	1,256 (51.6)	331 (54.6)	337 (54.8)	287 (47.5)	301 (49.3)
IV	164 (6.7)	68 (11.2)	39 (6.3)	34 (5.6)	23 (3.8)
Missing, n	276	72	53	78	73
WHO group I diagnosis, n (%)					
APAH–APAH–HIV	49 (1.8)	15 (2.2)	14 (2.1)	10 (1.5)	10 (1.5)
APAH–collagen vascular disease/connective tissue disease	708 (26.1)	180 (26.5)	178 (26.6)	176 (25.8)	174 (25.5)
APAH–congenital systemic-to-pulmonary shunts	271 (10.0)	25 (3.7)	48 (7.2)	77 (11.3)	121 (17.7)
APAH–drugs and toxins	155 (5.7)	50 (7.4)	47 (7.0)	35 (5.1)	23 (3.4)
APAH–other	33 (1.2)	10 (1.5)	9 (1.3)	8 (1.2)	6 (0.9)
APAH–portal hypertension	163 (6.0)	34 (5.0)	38 (5.7)	41 (6.0)	50 (7.3)
FPAH	78 (2.9)	23 (3.4)	21 (3.1)	19 (2.8)	15 (2.2)
IPAH	1,242 (45.8)	338 (49.9)	311 (46.6)	312 (45.7)	281 (41.1)
Pulmonary capillary hemangiomatosis	1 (0.04)	0	0	0	1 (0.1)
Pulmonary veno-occlusive disease	11 (0.4)	3 (0.4)	2 (0.3)	4 (0.6)	2 (0.3)

^a^Incident cases were defined as patients who received a diagnosis of PAH confirmed by RHC during study recruitment; prevalent cases were defined as patients diagnosed prior to the start of the study [24]. ^b^Patients were deemed newly diagnosed if the qualifying RHC was performed within the 3 months preceding enrollment to REVEAL, and previously diagnosed if the qualifying RHC was prior to the 3 months before enrollment [24]. APAH: associated PAH; BMI: body mass index; FC: functional class; FPAH: familial PAH; HIV: human immunodeficiency virus; IPAH: idiopathic PAH; IQR: interquartile range; NYHA: New York Heart Association; PAH: pulmonary arterial hypertension; PAPi: pulmonary artery pulsatility index; RHC: right heart catheterization; SD: standard deviation; WHO: World Health Organization.

**Table 2:** Clinical Characteristics by PAPi Quartile

Characteristic	PAPi quartile				
	Overall (N = 2,711)	< 3.55 (n = 678)	≥ 3.55 to < 5.5 (n = 668)	≥ 5.50 to < 9.0 (n = 682)	≥ 9.0 (n = 683)
Heart rate, bpm					
N	2,574	643	635	653	643
Mean (SD)	83.1 (14.7)	85.9 (15.1)	83.7 (14.5)	82.0 (14.5)	80.7 (14.3)
Median (IQR)	82.0 (72.0–93.0)	85.0 (76.0–96.0)	83.0 (72.0–93.5)	81.0 (72.0–92.0)	80.0 (70.0–90.0)
Missing, n	137	35	33	29	40
Systolic blood pressure, mm Hg					
N	2,593	647	636	657	653
Mean (SD)	117.1 (17.6)	115.5 (18.1)	117.6 (17.6)	117.7 (17.3)	117.5 (17.3)
Median (IQR)	115.0 (104.0–128.0)	113.0 (102.0–126.0)	116.0 (105.0–130.0)	115.0 (106.0–128.0)	118.0 (104.0–128.0)
Missing, n	118	31	32	25	30
Diastolic blood pressure, mm Hg					
N	2,591	647	636	657	651
Mean (SD)	70.2 (11.1)	70.7 (11.6)	70.0 (11.4)	70.3 (10.7)	69.9 (10.6)
Median (IQR)	70.0 (62.0–78.0)	70.0 (62.0–79.0)	70.0 (60.0–78.0)	70.0 (62.0–77.0)	70.0 (62.0–78.0)
Missing, n	120	31	32	25	32
6-min walk distance, m					
N	2,139	509	527	553	550
Mean (SD)	361.6 (127.5)	340.1 (126.0)	355.0 (129.1)	369.4 (131.4)	380.1 (120.0)
Median (IQR)	371.9 (278.9–446.8)	358.0 (256.0–428.0)	365.8 (271.3–441.0)	380.0 (288.3–451.1)	383.0 (310.9–454.0)
Missing, n	572	169	141	129	133
BNP value, pg/mL					
N	1,315	339	312	338	326
Mean (SD)	323.5 (616.6)	427.9 (789.0)	325.5 (550.9)	309.6 (643.8)	227.5 (385.6)
Median (IQR)	131.0 (45.0–367.5)	202.0 (66.0–505.0)	137.0 (44.0–379.8)	125.5 (46.3–298.0)	98.0 (37.0–272.3)
Missing, n	1,396	339	356	344	357
Most recent BNP, pg/mL					
N	279	74	83	64	58
Mean (SD)	2,205.8 (6,818.5)	2,002.7 (3,606.7)	3,909.5 (11,710.0)	1,382.1 (2,062.5)	935.7 (1,200.4)
Median (IQR)	613.0 (157.0–1,824.5)	1,026.5 (226.0–2,244.0)	613.0 (175.0–2,543.5)	530.5 (142.5–1,328.0)	551.5 (131.8–1,085.8)
Missing, n	2,432	604	585	618	625
PAH risk score (REVEAL 2.0 Risk Calculator)					
N	2,711	678	668	682	683
Mean (SD)	7.6 (2.3)	8.2 (2.4)	7.6 (2.3)	7.4 (2.4)	7.2 (2.1)
Median (IQR)	8.0 (6.0–9.0)	8.0 (7.0–10.0)	8.0 (6.0–9.0)	7.0 (6.0–9.0)	7.0 (6.0–8.0)
Baseline mPAP (at rest)					
N	2,654	665	649	670	670
Mean (SD)	49.6 (14.4)	48.6 (13.4)	49.4 (13.9)	50.5 (14.4)	49.7 (15.6)
Median (IQR)	48.0 (40.0–58.0)	48.0 (40.0–57.0)	48.0 (40.0–58.0)	50.0 (40.0–59.0)	48.0 (38.0–58.0)
Missing, n	57	13	19	12	13
Mixed venous O_2_ saturation					
N	1,741	433	422	442	444
Mean (SD)	63.8 (9.8)	58.5 (10.9)	63.7 (8.9)	66.0 (9.1)	66.9 (7.6)
Median (IQR)	65.0 (58.0–70.0)	59.0 (51.0–66.0)	64.0 (57.0–70.0)	66.0 (61.0–71.8)	67.5 (62.0–72.0)
Missing, n	970	245	246	240	239
Cardiac index (L/min/m^2^)					
N	2,193	569	544	542	538
Mean (SD)	2.4 (0.8)	2.2 (0.8)	2.4 (0.8)	2.5 (0.8)	2.5 (0.7)
Median (IQR)	2.3 (1.8–2.8)	2.0 (1.6–2.6)	2.3 (1.8–2.8)	2.4 (1.9–2.9)	2.5 (2.1–2.9)
Missing, n	518	109	124	140	145
PVR, Wood units					
N	2,516	652	621	632	611
Mean (SD)	10.3 (6.9)	10.9 (6.7)	10.0 (6.0)	10.1 (6.2)	10.2 (8.6)
Median (IQR)	8.9 (5.8–13.1)	9.8 (5.9–14.5)	8.6 (5.7–12.9)	8.7 (5.7–12.9)	8.6 (5.8–11.8)
Missing, n	195	26	47	50	72
Baseline PCWP (at rest), mm Hg					
N	2,585	645	633	659	648
Mean (SD)	9.8 (4.0)	10.7 (3.9)	10.5 (4.1)	9.7 (3.7)	8.2 (4.0)
Median (IQR)	10.0 (7.0–12.0)	11.0 (8.0–13.0)	10.0 (8.0–13.0)	9.0 (7.0–12.0)	8.0 (6.0–11.0)
Missing, n	126	33	35	23	35
Glomerular filtration rate at enrollment, mL/min/1.73 m^2^					
N	2,158	545	526	541	546
Mean (SD)	75.0 (26.5)	73.5 (26.2)	73.2 (27.3)	76.0 (24.9)	77.1 (27.2)
Median (IQR)	73.5 (56.7–93.5)	71.9 (54.2–91.6)	71.4 (54.6–92.2)	74.2 (59.3–92.2)	76.8 (58.3–96.2)
Missing, n	553	133	142	141	137
Borg dyspnea scale^a^					
N	1,922	452	484	501	485
Mean (SD)	3.0 (2.0)	3.1 (2.0)	3.3 (2.1)	3.0 (2.0)	2.8 (1.8)
Median (IQR)	3.0 (2.0–4.0)	3.0 (1.8–4.0)	3.0 (2.0–4.0)	3.0 (2.0–4.0)	3.0 (1.0–4.0)
Missing, n	789	226	184	181	198
Medical history of obstructive lung disease, n (%)					
Yes	386 (14.7)	90 (13.8)	105 (16.2)	107 (16.2)	84 (12.6)
No	2,243 (85.3)	561 (86.2)	544 (83.8)	553 (83.8)	585 (87.4)
Missing	82	27	19	22	14
Medical history of reactive airways disease, n (%)					
Yes	257 (9.9)	60 (9.3)	67 (10.4)	61 (9.4)	69 (10.4)
No	2,346 (90.1)	587 (90.7)	575 (89.6)	591 (90.6)	593 (89.6)
Missing	108	31	26	30	21
Medical history of sleep apnea, n (%)					
Yes	553 (21.6)	169 (26.9)	167 (26.2)	126 (19.7)	91 (14.0)
No	2,003 (78.4)	460 (73.1)	470 (73.8)	512 (80.3)	561 (86.0)
Missing	155	49	31	44	31
History of lung transplant, n (%)					
Yes	5 (0.2)	1 (0.1)	2 (0.3)	2 (0.3)	0
No	2,706 (99.8)	677 (99.9)	666 (99.7)	680 (99.7)	683 (100)
History of atrial septostomy, n (%)					
Yes	18 (0.7)	1 (0.1)	2 (0.3)	6 (0.9)	9 (1.3)
No	2,693 (99.3)	677 (99.9)	666 (99.7)	676 (99.1)	674 (98.7)
COPD, n (%)					
Yes	266 (10.0)	63 (9.4)	75 (11.5)	72 (10.7)	56 (8.3)
No	2,405 (90.0)	606 (90.6)	577 (88.5)	601 (89.3)	621 (91.7)
Missing	40	9	16	9	6
Pulmonary embolism, n (%)					
Yes	82 (3.1)	24 (3.6)	23 (3.5)	19 (2.8)	16 (2.4)
No	2,589 (96.9)	645 (96.4)	629 (96.5)	654 (97.2)	661 (97.6)
Missing	40	9	16	9	6
Congenital heart disease, n (%)					
Yes	271 (10.0)	25 (3.7)	48 (7.2)	77 (11.3)	121 (17.7)
No	2,440 (90.0)	653 (96.3)	620 (92.8)	605 (88.7)	562 (82.3)
Any prostacyclin, n (%)					
Yes	1,013 (38.1)	310 (46.5)	265 (40.8)	230 (34.3)	208 (31.0)
No	1,645 (61.9)	357 (53.5)	384 (59.2)	440 (65.7)	464 (69.0)
Missing	53	11	19	12	11
Any phosphodiesterase-5 inhibitor, n (%)					
Yes	1,239 (46.6)	300 (45.0)	294 (45.3)	328 (49.0)	317 (47.2)
No	1,419 (53.4)	367 (55.0)	355 (54.7)	342 (51.0)	355 (52.8)
Missing	53	11	19	12	11
Any endothelin receptor antagonist, n (%)					
Yes	1,118 (42.1)	259 (38.8)	241 (37.1)	307 (45.8)	311 (46.3)
No	1,540 (57.9)	408 (61.2)	408 (62.9)	363 (54.2)	361 (53.7)
Missing	53	11	19	12	11

^a^Scale ranges from 0, where breathing is causing no difficulty at all, through 10, where breathing difficulty is maximal. BNP: brain natriuretic peptide; bpm: beats per minute; COPD: chronic obstructive pulmonary disease; IQR: interquartile range; mPAP: mean pulmonary artery pressure; PAH: pulmonary arterial hypertension; PAPi: pulmonary artery pulsatility index; PCWP: pulmonary capillary wedge pressure; PVR: pulmonary vascular resistance; REVEAL: Registry to Evaluate Early and Long-Term PAH Disease Management; RHC: right heart catheterization; SD: standard deviation.

There was a trend for a longer 6MWD in the highest PAPi quartiles (Table 2). In additional analysis by absolute cut-off value < 3.55 versus ≥ 3.55, patients with PAPi values < 3.55 had a poorer 6MWD, a higher BNP, and a higher PAH risk score (per REVEAL 2.0 Risk Calculator) compared with patients with values ≥ 3.55 (Supplementary Material 2, cr.elmerpub.com). In addition, patients with PAPi values < 3.55 were more likely to receive prostacyclin therapy and less likely to receive endothelin receptor antagonist therapy than those with values ≥ 3.55 (Supplementary Material 2, cr.elmerpub.com).

The baseline and clinical characteristics of the incident and prevalent groups were similar (Supplementary Materials 3–6, cr.elmerpub.com), with the exception that a higher proportion of patients in the incident group than in the prevalent group had chronic obstructive pulmonary disease (overall 14.0% vs. 8.0%). Additionally, while more severe functional impairment for some characteristics (e.g., shorter 6MWD) was observed among lower PAPi quartiles in the incident group, this was not observed in the prevalent group (Supplementary Materials 3–6, cr.elmerpub.com).

### Hospitalization and mortality by PAPi quartile

**Figure 2:**
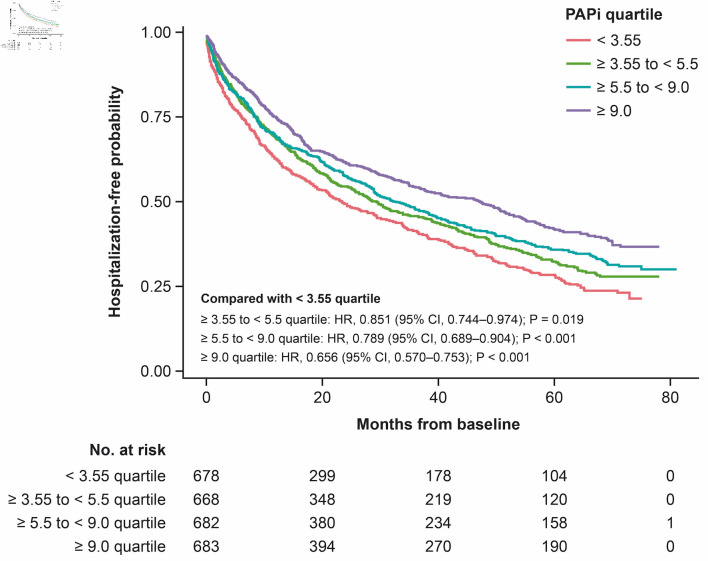
Probability of patients remaining hospitalization-free according to PAPi quartile. CI: confidence interval; HR: hazard ratio; PAPi: pulmonary artery pulsatility index.

Of 2,414 patients assessed for hospitalization, 1,326 (54.9%) were hospitalized during the study (March 2006 to December 2012) (Table 3). There was a significant difference between PAPi quartiles in the proportion of patients hospitalized; lower PAPi values were associated with increasing probability of hospitalization: 291 (48.0%) in the ≥ 9.0 quartile, 333 (54.8%) in the ≥ 5.5 to < 9.0 quartile, 340 (57.2%) in the ≥ 3.55 to < 5.5 quartile, and 362 (59.7%) in the < 3.55 quartile (P < 0.001). Compared with patients in the lowest quartile, there was a 34.4%, 21.1%, and 14.9% lower risk of hospitalization over the study period in the ≥ 9.0 (P < 0.001), ≥ 5.5 to < 9.0 (P < 0.001), and ≥ 3.55 to < 5.5 (P = 0.019) quartiles, respectively (Fig. 2).

**Table 3:** Hospitalization and Mortality by PAPi Quartile (36-Month Follow-Up)

	PAPi quartile					P value^a^
	Overall	< 3.55	≥ 3.55 to < 5.5	≥ 5.5 to < 9.0	≥ 9.0	
Hospitalization, n (%)	N = 2,414	n = 606	n = 594	n = 608	n = 606	
Yes	1,326 (54.9)	362 (59.7)	340 (57.2)	333 (54.8)	291 (48.0)	< 0.001
No	1,088 (45.1)	244 (40.3)	254 (42.8)	275 (45.2)	315 (52.0)	
Mortality, n (%)	N = 2,427	n = 613	n = 588	n = 603	n = 623	
Yes	681 (28.1)	220 (35.9)	157 (26.7)	154 (25.5)	150 (24.1)	< 0.001
No	1,746 (71.9)	393 (64.1)	431 (73.3)	449 (74.5)	473 (75.9)	

^a^Pearson’s Chi-squared test. PAPi: pulmonary artery pulsatility index.

**Figure 3:**
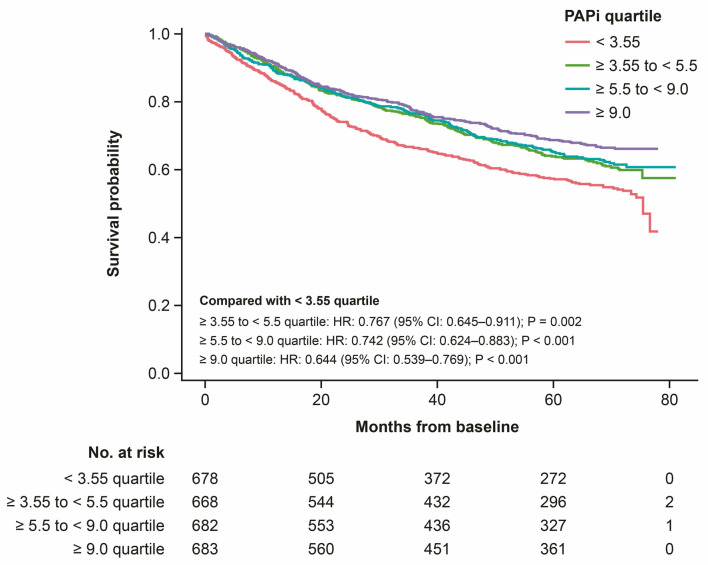
Mortality rates according to PAPi quartile. CI: confidence interval; HR: hazard ratio; PAPi: pulmonary artery pulsatility index.

A total of 681 (28.1%) patients died during the study (Table 3). A similar pattern to hospitalization was observed for mortality; there were 150 deaths (24.1%) in the ≥ 9.0 quartile, 154 (25.5%) in the ≥ 5.5 to < 9.0 quartile, 157 (26.7%) in the ≥ 3.55 to < 5.5 quartile, and 220 (35.9%) in the < 3.55 quartile (P < 0.001). There was a 35.6%, 25.8%, and 23.3% reduction in mortality over the study period in the ≥ 9.0 (P < 0.001), ≥ 5.5 to < 9.0 (P < 0.001), and ≥ 3.55 to < 5.5 (P = 0.002) quartiles, versus the < 3.55 quartile, respectively (Fig. 3).

Analysis of hospitalization and mortality in the incident and prevalent groups demonstrated a similar pattern to the overall analysis, with patients in higher PAPi quartiles experiencing lower rates of hospitalization and death (Supplementary Materials 7, 8, cr.elmerpub.com). However, in patients with prevalent PAH, there was no significant difference between the < 3.55 quartile and the ≥ 3.55 to < 5.5 quartile (Supplementary Materials 7, 8, cr.elmerpub.com).

### Optimal cut-off value for PAPi risk categories

**Figure 4:**
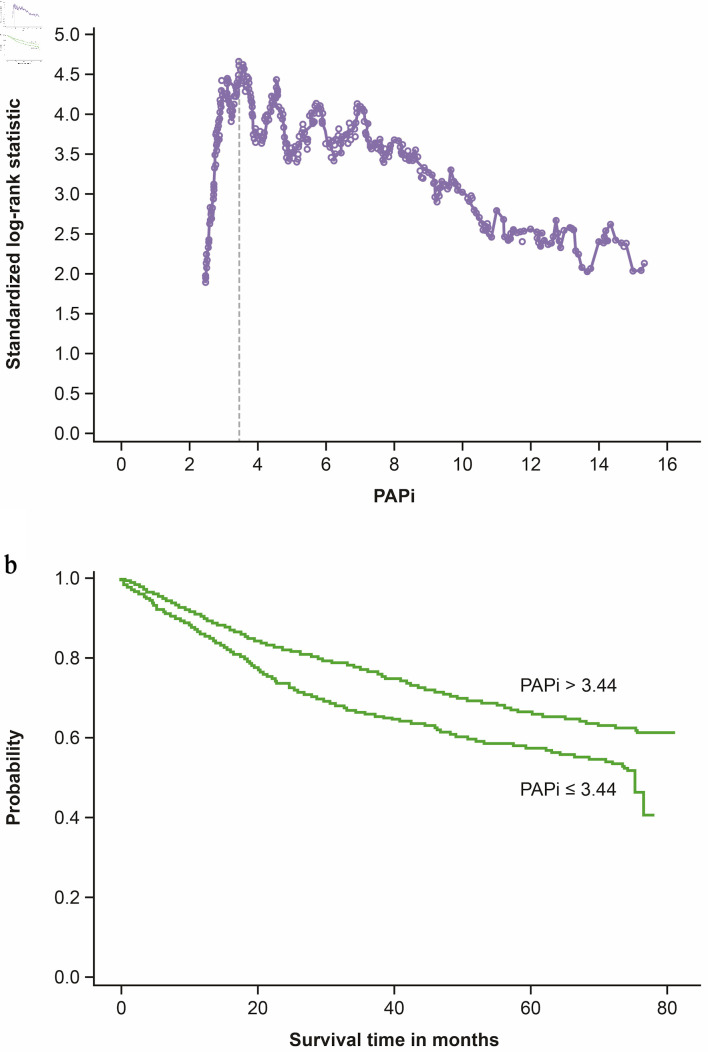
PAPi cut-off point for mortality using log-rank statistics. (a) The optimal cut-off point was 3.44. (b) The corresponding PAPi groups (≤ 3.44 and > 3.44) provided the best risk discrimination (P < 0.001). PAPi: pulmonary artery pulsatility index.

Standardized log-rank statistics of all possible PAPi cut-off points were generated to determine the optimal cut-off point associated with mortality. The optimal cut-off point based on maximally selected log-rank statistics was 3.44 for PAPi (Fig. 4a). Kaplan–Meier curves for the corresponding PAPi groups (≤ 3.44 and > 3.44) provided the best risk discrimination (P < 0.001) (Fig. 4b). Additionally, the range of PAPi values from 0 to 56 validated in the histogram distribution (Supplementary Material 9, cr.elmerpub.com) was in line with prior research in a smaller sample of patients with PAH [22].

## Discussion

This large study of 2,711 patients in REVEAL demonstrated that PAPi is a useful prognostic tool in patients with PAH. In the analysis by PAPi quartile, lower PAPi values (< 3.55 and ≥ 3.55 to < 5.5) were associated with more severe functional impairment, greater risk of hospitalization, and a worse mortality rate compared with higher PAPi values (≥ 5.5 to < 9.0 and ≥ 9.0). Notably, Kaplan–Meier curves for both hospitalization and mortality began to diverge almost immediately. Another study showed that lower PAPi quartile was associated with worse mortality and hospitalization outcomes in all patients undergoing RHC [19], supporting the current study’s results that quartile data are more robust than a binary value. A study of the therapies received by patients who deteriorated to NYHA/WHO functional class IV or had died indicated that many were not being treated as aggressively as recommended by guidelines (i.e., with systemic prostacyclin and/or combination therapy) [26]. Although early combination therapy was not widely used at the beginning of REVEAL, the PAPi cut-off association with mortality and hospitalization was demonstrated over 5 years—a long follow-up period.

The analysis of baseline characteristics by absolute cut-off PAPi value of < 3.55 versus ≥ 3.55 showed a slight difference between the two groups in 6MWD, BNP, and PAH risk score (per REVEAL 2.0 Risk Calculator), which was not observed in the analysis by PAPi quartile. The study identified a PAPi cut-off of 3.55 as a good predictor of clinical worsening (6MWD, functional class, and BNP). While the value of 3.6 has previously been validated as a PAPi cut-off [20, 21], 3.55 was chosen for our analysis because it provides a more precise cut-off for examining potential differences in baseline characteristics compared with values below and above 3.55.

PAPi is widely used in clinical decision-making in cardiac critical care [20]. A recent study demonstrated that PAPi is associated with mortality and cardiac outcomes across a range of cardiovascular diseases, with patients in the lowest PAPi quartile being at the highest risk for all-cause mortality and adverse cardiovascular-related outcomes [19]. PAPi has also been shown to predict RV failure in patients with inferior myocardial infarction and left ventricular assist device implantation [16–18]. Three studies have evaluated the utility of PAPi as a prognostic tool in patients with PAH [13, 22, 23]. However, all the patients in these studies had primary pulmonary hypertension (now called idiopathic PAH); other PAH subgroups were not included. A registry study of 102 patients with PAH enrolled between 2003 and 2016 in Singapore found that patients with PAPi values < 5.3 had an almost three times higher risk of mortality than patients with PAPi values ≥ 5.3 [23]. In that study, RAP was the component of PAPi that had the strongest prognostic value [23]. Another study using data in the US National Institutes of Health Registry for Primary Pulmonary Hypertension from 272 patients also found that mortality was higher in patients with a PAPi value in the lowest quartile (< 3.7) compared with values in higher quartiles (≥ 3.7; 67.2% vs. 41.5% over 3 years) [22]. A retrospective cohort study of 590 patients in Canada between 2016 and 2020 found that PAPi values < 5.3 were associated with higher 1-year mortality compared with values ≥ 5.3 (10.2% vs. 5.2%) [13]. In this study, as previously noted in the study from Singapore [23], the RAP component of PAPi predicted mortality as accurately as PAPi overall [13].

Although these studies were small, they do provide some evidence that quartiles may be the best use of PAPi as opposed to binary cut-offs. As with the current study, they show a clear trend for an association of higher PAPi quartiles with lower morbidity and mortality; the quartile groups were also similar compared with the binary cut-offs. For the present study, exploration of the binary cut-offs was included in the analysis due to prior research conducted on PAPi in the evaluation of PAH.

This study has limitations. REVEAL recruited patients between 2006 and 2012, before early combination therapy became widely used for patients with PAH. Hospitalization and mortality data from our study should therefore be considered in the context of changing management practices over the years. It was also beyond the scope of our analysis to evaluate whether any single component of the PAPi was driving its prognostic value, as has been found previously [13, 23]. A key strength of the study was the large number of patients (N = 2,711)—a much larger cohort than evaluated in other similar studies.

Based on this large cohort of patients from REVEAL, PAPi was a good prognostic tool and long-term predictor of hospitalization and mortality in PAH. In addition, using quartiles seemed more sensitive than using binary cut-offs. PAPi may be an additional metric to improve prediction of outcomes in PAH alone or added to other risk assessment tools. Future studies should longitudinally investigate whether PAPi demonstrates sustained prognostic utility of baseline hemodynamic measures in the current era of early and effective combination therapy.

## Supplementary Material

Suppl 1 Baseline characteristics by PAPi cut-off value < 3.55 versus ≥ 3.55.

Suppl 2 Clinical characteristics by PAPi cut-off value < 3.55 versus ≥ 3.55.

Suppl 3 Baseline characteristics by PAPi quartile: prevalent group.

Suppl 4 Baseline characteristics by PAPi quartile: incident group.

Suppl 5 Clinical characteristics by PAPi quartile: prevalent group.

Suppl 6 Clinical characteristics by PAPi quartile: incident group.

Suppl 7 Probability of patients remaining hospitalization-free according to PAPi quartile (incident and prevalent patients).

Suppl 8 Mortality according to PAPi quartile (incident and prevalent patients).

Suppl 9 Histogram of PAPi scores/values.

## Data Availability

The data sharing policy of Johnson & Johnson is available at https://innovativemedicine.jnj.com/our-innovation/clinical-trials/transparency. As noted on this site, requests for access to the study data can be submitted through the Yale Open Data Access (YODA) Project site at http://yoda.yale.edu.

## Abbreviations

6MWD: 6-min walk distance
APAH: associated pulmonary arterial hypertension
BMI: body mass index
BNP: brain natriuretic peptide
bpm: beats per minute
CI: confidence interval
COPD: chronic obstructive pulmonary disease
ERS: European Respiratory Society
ESC: European Society of Cardiology
FC: functional class
FPAH: familial pulmonary arterial hypertension
HIV: human immunodeficiency virus
HR: hazard ratio
IPAH: idiopathic pulmonary arterial hypertension
IQR: interquartile range
mPAP: mean pulmonary artery pressure
NYHA: New York Heart Association
PA: pulmonary arterial
PAH: pulmonary arterial hypertension
PAPi: pulmonary artery pulsatility index
PAWP: pulmonary artery wedge pressure
PCWP: pulmonary capillary wedge pressure
PVR: pulmonary vascular resistance
RAP: right atrial pressure
REVEAL: Registry to Evaluate Early and Long-Term PAH Disease Management
RHC: right heart catheterization
RV: right ventricular
RV-PA: right ventricular-pulmonary arterial
SD: standard deviation
WHO: World Health Organization
