# Supplementary material for: Predictive Value of the Pulmonary Artery Pulsatility Index in Pulmonary Arterial Hypertension: REVEAL Analysis
Source: Cardiol Res. 2026 Jun 5;17(3):214–26. doi: 10.14740/cr2225 (PMC13278699; doi:10.14740/cr2225)
Supplement: Suppl 1 — Baseline characteristics by PAPi cut-off value < 3.55 versus ≥ 3.55. [file cr-17-03-214-s001.docx]

**Suppl 1.** Baseline Characteristics by PAPi Cut-off Value < 3.55 Versus ≥ 3.55

|  | **PAPi Value** | | | |
| --- | --- | --- | --- | --- |
| **Characteristic** | **Overall (N = 2,711)** | **< 3.55 (n = 678)** | **≥ 3.55 (n = 2,033)** | |
| Age, years |  |  |  | |
| n | 2,711 | 678 | 2,033 | |
| Mean (SD) | 52.4 (14.7) | 51.4 (13.9) | 52.8 (15.0) | |
| Median (IQR) | 53.0 (42.4-63.3) | 51.4 (41.6-61.3) | 53.6 (42.7-63.8) | |
| Age at diagnosis, years |  |  |  | |
| n | 2,711 | 678 | 2,033 | |
| Mean (SD) | 49.7 (15.5) | 49.1 (14.2) | 49.9 (16.0) | |
| Median (IQR) | 50.2 (39.0-61.1) | 49.4 (38.7-59.2) | 50.5 (39.0-61.8) | |
| Sex, n (%) |  |  |  | |
| Male | 561 (20.7) | 164 (24.2) | 397 (19.5) | |
| Female | 2,150 (79.3) | 514 (75.8) | 1,636 (80.5) | |
| Race, n (%) |  |  |  | |
| White | 1,969 (72.6) | 480 (70.8) | 1,489 (73.2) | |
| Black | 347 (12.8) | 112 (16.5) | 235 (11.6) | |
| Hispanic | 236 (8.7) | 48 (7.1) | 188 (9.2) | |
| Asian | 86 (3.2) | 17 (2.5) | 69 (3.4) | |
| Other | 73 (2.7) | 21 (3.1) | 52 (2.6) | |
| BMI, kg/m^2^ |  |  |  | |
| n | 2,587 | 640 | 1,947 | |
| Mean (SD) | 28.3 (7.0) | 29.3 (7.4) | 27.9 (6.8) | |
| Median (IQR) | 27.1 (23.2-31.8) | 28.4 (24.2-33.1) | 26.8 (23.0-31.5) | |
| Missing, n | 124 | 38 | 86 | |
| PAH Diagnosis, n (%)^a^ |  |  |  | |
| Incident | 900 (33.2) | 248 (36.6) | 652 (32.1) | |
| Prevalent | 1,811 (66.8) | 430 (63.4) | 1,381 (67.9) | |
| Diagnostic status, n (%)^b^ |  |  |  | |
| Newly diagnosed | 737 (27.2) | 206 (30.4) | 531 (26.1) | |
| Previously diagnosed | 1,974 (72.8) | 472 (69.6) | 1,502 (73.9) | |
| NYHA/WHO FC, n (%) |  |  |  | |
| I | 172 (7.1) | 26 (4.3) | 146 (8.0) | |
| II | 843 (34.6) | 181 (29.9) | 662 (36.2) | |
| III | 1,256 (51.6) | 331 (54.6) | 925 (50.6) | |
| IV | 164 (6.7) | 68 (11.2) | 96 (5.2) | |
| Missing | 276 | 72 | 204 | |
| WHO Group I diagnosis, n (%) |  |  |  | |
| APAH – APAH – HIV | 49 (1.8) | 15 (2.2) | 34 (1.7) | |
| APAH – Collagen vascular disease/connective tissue disease | 708 (26.1) | 180 (26.5) | 528 (26.0) | |
| APAH – Congenital systemic-to-pulmonary shunts | 271 (10.0) | 25 (3.7) | 246 (12.1) | |
| APAH – Drugs and toxins | 155 (5.7) | 50 (7.4) | 105 (5.2) | |
| APAH – Other | 33 (1.2) | 10 (1.5) | 23 (1.1) | |
| APAH – Portal hypertension | 163 (6.0) | 34 (5.0) | 129 (6.3) | |
| FPAH | 78 (2.9) | 23 (3.4) | 55 (2.7) | |
| IPAH | 1,242 (45.8) | 338 (49.9) | 904 (44.5) | |
| Pulmonary capillary hemangiomatosis | 1 (0.04) | 0 | 1 (0.05) | |
| Pulmonary veno-occlusive disease | 11 (0.4) | 3 (0.4) | 8 (0.4) | |
| ^a^Incident cases were defined as patients who received a diagnosis of PAH confirmed by RHC during study recruitment; prevalent cases were defined as patients diagnosed prior to the start of the study [1].  ^b^Patients were deemed newly diagnosed if the qualifying RHC was performed within the 3 months preceding enrollment to REVEAL, and previously diagnosed if the qualifying RHC was prior to the 3 months before enrollment [1].  APAH: associated PAH; BMI: body mass index; FC: functional class; FPAH: familial PAH; HIV: human immunodeficiency virus; IPAH: idiopathic PAH; IQR: interquartile range; NYHA: New York Heart Association; PAH: pulmonary arterial hypertension; PAPi: pulmonary artery pulsatility index; REVEAL: Registry to Evaluate Early and Long-Term PAH Disease Management; RHC: right heart catheterization; SD: standard deviation; WHO: World Health Organization. | | | |  |
|  | | | |  |

**Reference**

1. McGoon MD, Miller DP. REVEAL: a contemporary US pulmonary arterial hypertension registry. Eur Respir Rev. 2012;21(123):8-18.
