# Supplementary material for: Predictive Value of the Pulmonary Artery Pulsatility Index in Pulmonary Arterial Hypertension: REVEAL Analysis
Source: Cardiol Res. 2026 Jun 5;17(3):214–26. doi: 10.14740/cr2225 (PMC13278699; doi:10.14740/cr2225)
Supplement: Suppl 2 — Clinical characteristics by PAPi cut-off value < 3.55 versus ≥ 3.55. [file cr-17-03-214-s002.docx]

**Suppl 2.** Clinical Characteristics by PAPi Cut-off Value < 3.55 Versus ≥ 3.55

|  | **PAPi Value** | | |
| --- | --- | --- | --- |
| **Characteristic** | **Overall (N = 2,711)** | **< 3.55 (n = 678)** | **≥ 3.55 (n = 2,033)** |
| Heart rate at time of vital signs, bpm |  |  |  |
| n | 2,574 | 643 | 1,931 |
| Mean (SD) | 83.1 (14.7) | 85.9 (15.1) | 82.1 (14.5) |
| Median (IQR) | 82.0 (72.0-93.0) | 85.0 (76.0-96.0) | 81.0 (72.0-92.0) |
| Missing, n | 137 | 35 | 102 |
| Systolic blood pressure, mmHg |  |  |  |
| n | 2,593 | 647 | 1,946 |
| Mean (SD) | 117.1 (17.6) | 115.5 (18.1) | 117.6 (17.4) |
| Median (IQR) | 115.0 (104.0-128.0) | 113.0 (102.0-126.0) | 116.0 (105.0-129.0) |
| Missing, n | 118 | 31 | 87 |
| Diastolic blood pressure, mmHg |  |  |  |
| n | 2,591 | 647 | 1,944 |
| Mean (SD) | 70.2 (11.1) | 70.7 (11.6) | 70.1 (10.9) |
| Median (IQR) | 70.0 (62.0-78.0) | 70.0 (62.0-79.0) | 70.0 (62.0-78.0) |
| Missing, n | 120 | 31 | 89 |
| Most recent 6-minute walk distance test, m |  |  |  |
| n | 2,139 | 509 | 1,630 |
| Mean (SD) | 361.6 (127.5) | 340.1 (126.0) | 368.4 (127.2) |
| Median (IQR) | 371.9 (278.9-446.8) | 358.0 (256.0-428.0) | 376.1 (289.7-451.0) |
| Missing, n | 572 | 169 | 403 |
| BNP Value, pg/mL |  |  |  |
| n | 1,315 | 339 | 976 |
| Mean (SD) | 323.5 (616.6) | 427.9 (789.0) | 287.2 (539.9) |
| Median (IQR) | 131.0 (45.0-367.5) | 202.0 (66.0-505.0) | 119.5 (41.8-306.0) |
| Missing, n | 1,396 | 339 | 1,057 |
| Most recent BNP, pg/mL |  |  |  |
| n | 279 | 74 | 205 |
| Mean (SD) | 2,205.8 (6,818.5) | 2,002.7 (3,606.7) | 2,279.1 (7,660.4) |
| Median (IQR) | 613.0 (157.0-1,824.5) | 1026.5 (226.0-2,244.0) | 552.0 (148.0-1,763.0) |
| Missing, n | 2,432 | 604 | 1,828 |
| PAH Risk score (REVEAL 2.0 Risk Calculator) |  |  |  |
| n | 2,711 | 678 | 2,033 |
| Mean (SD) | 7.6 (2.3) | 8.2 (2.4) | 7.4 (2.3) |
| Median (IQR) | 8.0 (6.0-9.0) | 8.0 (7.0-10.0) | 7.0 (6.0-9.0) |
| Most recent baseline mPAP at rest, entered or calculated (range restricted), mmHg | | | |
| n | 2,654 | 665 | 1,989 |
| Mean (SD) | 49.6 (14.4) | 48.6 (13.4) | 49.9 (14.7) |
| Median (IQR) | 48.0 (40.0-58.0) | 48.0 (40.0-57.0) | 49.0 (40.0-58.0) |
| Missing, n | 57 | 13 | 44 |
| Actual most recent mixed venous O_2_ saturation |  |  |  |
| n | 1,741 | 433 | 1,308 |
| Mean (SD) | 63.8 (9.8) | 58.5 (10.9) | 65.6 (8.7) |
| Median (IQR) | 65.0 (58.0-70.0) | 59.0 (51.0-66.0) | 66.0 (61.0-71.0) |
| Missing, n | 970 | 245 | 725 |
| Most recent cardiac index (minimum output – not preferred analysis variable), L/min/m^2^ | | | |
| n | 2,193 | 569 | 1,624 |
| Mean (SD) | 2.4 (0.8) | 2.2 (0.8) | 2.5 (0.8) |
| Median (IQR) | 2.3 (1.8-2.8) | 2.0 (1.6-2.6) | 2.4 (1.9-2.9) |
| Missing, n | 518 | 109 | 409 |
| Most recent PVR (Fick hierarchy – preferred analysis variable), Wood units | | | |
| n | 2,516 | 652 | 1,864 |
| Mean (SD) | 10.3 (6.9) | 10.9 (6.7) | 10.1 (7.0) |
| Median (IQR) | 8.9 (5.8-13.1) | 9.8 (5.9-14.5) | 8.6 (5.7-12.6) |
| Missing, n | 195 | 26 | 169 |
| Most recent baseline PCWP, at rest (range restricted), mmHg |  |  |  |
| n | 2,585 | 645 | 1,940 |
| Mean (SD) | 9.8 (4.0) | 10.7 (3.9) | 9.5 (4.0) |
| Median (IQR) | 10.0 (7.0-12.0) | 11.0 (8.0-13.0) | 9.0 (7.0-12.0) |
| Missing, n | 126 | 33 | 93 |
| Glomerular filtration rate at enrollment, mL/min/1.73 m^2^ |  |  |  |
| n | 2,158 | 545 | 1,613 |
| Mean (SD) | 75.0 (26.5) | 73.5 (26.2) | 75.5 (26.6) |
| Median (IQR) | 73.5 (56.7-93.5) | 71.9 (54.2-91.6) | 74.0 (57.4-94.2) |
| Missing, n | 553 | 133 | 420 |
| Borg Dyspnea Scale^a^ |  |  |  |
| n | 1,922 | 452 | 1,470 |
| Mean (SD) | 3.0 (2.0) | 3.1 (2.0) | 3.0 (1.9) |
| Median (IQR) | 3.0 (2.0-4.0) | 3.0 (1.8-4.0) | 3.0 (2.0-4.0) |
| Missing, n | 789 | 226 | 563 |
| Medical history of obstructive lung disease, n (%) |  |  |  |
| Yes | 386 (14.7) | 90 (13.8) | 296 (15.0) |
| No | 2,243 (85.3) | 561 (86.2) | 1,682 (85.0) |
| Missing | 82 | 27 | 55 |
| Medical history of reactive airways disease, n (%) |  |  |  |
| Yes | 257 (9.9) | 60 (9.3) | 197 (10.1) |
| No | 2,346 (90.1) | 587 (90.7) | 1,759 (89.9) |
| Missing | 108 | 31 | 77 |
| Medical history of sleep apnea, n (%) |  |  |  |
| Yes | 553 (21.6) | 169 (26.9) | 384 (19.9) |
| No | 2,003 (78.4) | 460 (73.1) | 1,543 (80.1) |
| Missing | 155 | 49 | 106 |
| History of lung transplant, n (%) |  |  |  |
| Yes | 5 (0.2) | 1 (0.1) | 4 (0.2) |
| No | 2,706 (99.8) | 677 (99.9) | 2,029 (99.8) |
| History of atrial septostomy, n (%) |  |  |  |
| Yes | 18 (0.7) | 1 (0.1) | 17 (0.8) |
| No | 2,693 (99.3) | 677 (99.9) | 2,016 (99.2) |
| COPD, n (%) |  |  |  |
| Yes | 266 (10.0) | 63 (9.4) | 203 (10.1) |
| No | 2,405 (90.0) | 606 (90.6) | 1,799 (89.9) |
| Missing | 40 | 9 | 31 |
| Pulmonary embolism, n (%) |  |  |  |
| Yes | 82 (3.1) | 24 (3.6) | 58 (2.9) |
| No | 2,589 (96.9) | 645 (96.4) | 1,944 (97.1) |
| Missing | 40 | 9 | 31 |
| Congenital heart disease, n (%) |  |  |  |
| Yes | 271 (10.0) | 25 (3.7) | 246 (12.1) |
| No | 2,440 (90.0) | 653 (96.3) | 1,787 (87.9) |
| Any prostacyclin, n (%) |  |  |  |
| Yes | 1,013 (38.1) | 310 (46.5) | 703 (35.3) |
| No | 1,645 (61.9) | 357 (53.5) | 1,288 (64.7) |
| Missing | 53 | 11 | 42 |
| Any phosphodiesterase-5 inhibitor, n (%) |  |  |  |
| Yes | 1,239 (46.6) | 300 (45.0) | 939 (47.2) |
| No | 1,419 (53.4) | 367 (55.0) | 1,052 (52.8) |
| Missing | 53 | 11 | 42 |
| Any endothelin receptor antagonist, n (%) |  |  |  |
| Yes | 1,118 (42.1) | 259 (38.8) | 859 (43.1) |
| No | 1,540 (57.9) | 408 (61.2) | 1,132 (56.9) |
| Missing | 53 | 11 | 42 |

^a^Scale ranges from 0, where breathing is causing no difficulty at all, through 10, where breathing difficulty is maximal.
BNP: brain natriuretic peptide; bpm: beats per minute; COPD: chronic obstructive pulmonary disease; IQR: interquartile range; mPAP: mean pulmonary artery pressure; PAH: pulmonary arterial hypertension; PAPi: pulmonary artery pulsatility index; PCWP: pulmonary capillary wedge pressure; PVR: pulmonary vascular resistance; REVEAL: Registry to Evaluate Early and Long-Term PAH Disease Management; SD: standard deviation.
