# Supplementary material for: Predictive Value of the Pulmonary Artery Pulsatility Index in Pulmonary Arterial Hypertension: REVEAL Analysis
Source: Cardiol Res. 2026 Jun 5;17(3):214–26. doi: 10.14740/cr2225 (PMC13278699; doi:10.14740/cr2225)
Supplement: Suppl 3 — Baseline characteristics by PAPi quartile: prevalent group. [file cr-17-03-214-s003.docx]

**Suppl 3.** Baseline Characteristics by PAPi Quartile: Prevalent Group

|  | **PAPi Quartile** | | | | |
| --- | --- | --- | --- | --- | --- |
| **Characteristic** | **Overall (N = 1,811)** | **< 3.55 (n = 430)** | **≥ 3.55 to < 5.5 (n = 425)** | **≥ 5.50 to < 9.0 (n = 475)** | **≥ 9.0 (n = 481)** |
| Age, years |  |  |  |  |  |
| n | 1,811 | 430 | 425 | 475 | 481 |
| Mean (SD) | 51.8 (14.3) | 51.0 (13.4) | 52.8 (13.7) | 51.7 (14.6) | 51.6 (15.2) |
| Median (IQR) | 52.3 (42.1-62.3) | 51.3 (41.2-60.3) | 53.8 (43.9-63.4) | 52.2 (41.4-62.3) | 51.8 (41.4-62.6) |
| Age at diagnosis, years | | | | | |
| n | 1,811 | 430 | 425 | 475 | 481 |
| Mean (SD) | 47.7 (15.2) | 47.5 (13.7) | 49.0 (14.6) | 47.5 (15.5) | 47.0 (16.7) |
| Median (IQR) | 48.6 (37.7-58.4) | 47.9 (38.0-56.7) | 49.3 (40.1-58.8) | 48.6 (36.9-58.2) | 47.7 (35.3-59.1) |
| Sex, n (%) |  |  |  |  |  |
| Male | 358 (19.8) | 95 (22.1) | 77 (18.1) | 97 (20.4) | 89 (18.5) |
| Female | 1,453 (80.2) | 335 (77.9) | 348 (81.9) | 378 (79.6) | 392 (81.5) |
| Race, n (%) |  |  |  |  |  |
| White | 1,320 (72.9) | 308 (71.6) | 313 (73.6) | 360 (75.8) | 339 (70.5) |
| Black | 227 (12.5) | 65 (15.1) | 58 (13.6) | 53 (11.2) | 51 (10.6) |
| Hispanic | 167 (9.2) | 33 (7.7) | 32 (7.5) | 42 (8.8) | 60 (12.5) |
| Asian | 61 (3.4) | 13 (3.0) | 15 (3.5) | 11 (2.3) | 22 (4.6) |
| Other | 36 (2.0) | 11 (2.6) | 7 (1.6) | 9 (1.9) | 9 (1.9) |
| BMI, kg/m^2^ |  |  |  |  |  |
| n | 1,740 | 412 | 405 | 457 | 466 |
| Mean (SD) | 28.0 (6.8) | 29.1 (7.3) | 28.7 (7.1) | 28.1 (6.8) | 26.2 (5.6) |
| Median (IQR) | 26.9 (23.1-31.4) | 28.1 (24.0-32.9) | 27.5 (23.2-32.6) | 27.0 (23.2-31.1) | 25.7 (21.9-29.4) |
| Missing, n | 71 | 18 | 20 | 18 | 15 |
| PAH Diagnosis, n (%) | | | | | |
| Prevalent | 1,811 (100) | 430 (100) | 425 (100) | 475 (100) | 481 (100) |
| Diagnostic status, n (%) | | | | | |
| Previously diagnosed^a^ | 1,811 (100) | 430 (100) | 425 (100) | 475 (100) | 481 (100) |
| NYHA/WHO FC, n (%) | | | | | |
| I | 141 (8.5) | 22 (5.6) | 34 (8.5) | 45 (10.5) | 40 (9.1) |
| II | 654 (39.4) | 142 (35.9) | 144 (36.1) | 184 (43.0) | 184 (42.0) |
| III | 797 (48.0) | 209 (52.9) | 202 (50.6) | 182 (42.5) | 204 (46.6) |
| IV | 68 (4.1) | 22 (5.6) | 19 (4.8) | 17 (4.0) | 10 (2.3) |
| Missing, n | 151 | 35 | 26 | 47 | 43 |
| WHO Group I diagnosis, n (%) | | | | | |
| APAH – APAH – HIV | 34 (1.9) | 9 (2.1) | 10 (2.4) | 8 (1.7) | 7 (1.5) |
| APAH – Collagen vascular disease/connective tissue disease | 430 (23.7) | 106 (24.7) | 110 (25.9) | 107 (22.5) | 107 (22.2) |
| APAH – Congenital systemic-to-pulmonary shunts | 226 (12.5) | 19 (4.4) | 37 (8.7) | 67 (14.1) | 103 (21.4) |
| APAH – Drugs and toxins | 114 (6.3) | 37 (8.6) | 37 (8.7) | 24 (5.1) | 16 (3.3) |
| APAH – Other | 23 (1.3) | 8 (1.9) | 4 (0.9) | 6 (1.3) | 5 (1.0) |
| APAH – Portal hypertension | 98 (5.4) | 18 (4.2) | 19 (4.5) | 30 (6.3) | 31 (6.4) |
| FPAH | 56 (3.1) | 20 (4.7) | 13 (3.1) | 15 (3.2) | 8 (1.7) |
| IPAH | 824 (45.5) | 213 (49.5) | 195 (45.9) | 215 (45.3) | 201 (41.8) |
| Pulmonary capillary hemangiomatosis | 1 (0.05) | 0 | 0 | 0 | 1 (0.2) |
| Pulmonary veno-occlusive disease | 5 (0.3) | 0 | 0 | 3 (0.6) | 2 (0.4) |

^a^Patients were deemed newly diagnosed if the qualifying RHC was performed within the 3 months preceding enrollment to REVEAL, and previously diagnosed if the qualifying RHC was prior to the 3 months before enrollment.
APAH: associated PAH; BMI: body mass index; FC: functional class; FPAH: familial PAH; HIV: human immunodeficiency virus; IPAH: idiopathic PAH; IQR: interquartile range; NYHA: New York Heart Association; PAH: pulmonary arterial hypertension; PAPi: pulmonary artery pulsatility index; REVEAL: Registry to Evaluate Early and Long-Term PAH Disease Management; RHC: right heart catheterization; SD: standard deviation; WHO: World Health Organization.
