# Supplementary material for: Predictive Value of the Pulmonary Artery Pulsatility Index in Pulmonary Arterial Hypertension: REVEAL Analysis
Source: Cardiol Res. 2026 Jun 5;17(3):214–26. doi: 10.14740/cr2225 (PMC13278699; doi:10.14740/cr2225)
Supplement: Suppl 4 — Baseline characteristics by PAPi quartile: incident group. [file cr-17-03-214-s004.docx]

**Suppl 4.** Baseline Characteristics by PAPi Quartile: Incident Group

|  | **PAPi Quartile** | | | | |
| --- | --- | --- | --- | --- | --- |
| **Characteristic** | **Overall (N = 900)** | **< 3.55 (n = 248)** | **≥ 3.55 to < 5.5 (n = 243)** | **≥ 5.5 to < 9.0 (n = 207)** | **≥ 9.0 (n = 202)** |
| Age, years |  |  |  |  |  |
| n | 900 | 248 | 243 | 207 | 202 |
| Mean (SD) | 53.8 (15.4) | 51.9 (14.6) | 54.7 (15.4) | 56.3 (15.1) | 52.4 (16.2) |
| Median (IQR) | 54.6 (43.2-65.3) | 51.4 (42.0-62.4) | 55.7 (44.9-65.0) | 57.1 (45.1-67.9) | 54.4 (40.4-64.4) |
| Age at diagnosis, years |  |  |  |  |  |
| n | 900 | 248 | 243 | 207 | 202 |
| Mean (SD) | 53.7 (15.4) | 51.8 (14.6) | 54.6 (15.4) | 56.2 (15.1) | 52.3 (16.2) |
| Median (IQR) | 54.4 (43.1-65.2) | 51.3 (42.0-62.3) | 55.6 (44.7-64.8) | 56.9 (44.9-67.9) | 54.2 (40.2-64.4) |
| Sex, n (%) |  |  |  |  |  |
| Male | 203 (22.6) | 69 (27.8) | 61 (25.1) | 35 (16.9) | 38 (18.8) |
| Female | 697 (77.4) | 179 (72.2) | 182 (74.9) | 172 (83.1) | 164 (81.2) |
| Race, n (%) |  |  |  |  |  |
| White | 649 (72.1) | 172 (69.4) | 176 (72.4) | 158 (76.3) | 143 (70.8) |
| Black | 120 (13.3) | 47 (19.0) | 31 (12.8) | 19 (9.2) | 23 (11.4) |
| Hispanic | 69 (7.7) | 15 (6.0) | 18 (7.4) | 18 (8.7) | 18 (8.9) |
| Asian | 25 (2.8) | 4 (1.6) | 8 (3.3) | 2 (1.0) | 11 (5.4) |
| Other | 37 (4.1) | 10 (4.0) | 10 (4.1) | 10 (4.8) | 7 (3.5) |
| BMI, kg/m^2^ |  |  |  |  |  |
| n | 847 | 228 | 232 | 194 | 193 |
| Mean (SD) | 28.8 (7.3) | 29.8 (7.5) | 30.3 (7.8) | 27.5 (6.0) | 27.3 (7.1) |
| Median (IQR) | 27.8 (23.7-32.7) | 29.0 (24.3-33.8) | 29.2 (25.1-34.9) | 26.6 (23.3-31.1) | 25.9 (21.7-31.2) |
| Missing, n | 53 | 20 | 11 | 13 | 9 |
| PAH Diagnosis, n (%) |  |  |  |  |  |
| Incident | 900 (100) | 248 (100) | 243 (100) | 207 (100) | 202 (100) |
| Diagnostic status, n (%)^a^ |  |  |  |  |  |
| Newly diagnosed | 737 (81.9) | 206 (83.1) | 199 (81.9) | 165 (79.7) | 167 (82.7) |
| Previously diagnosed | 163 (18.1) | 42 (16.9) | 44 (18.1) | 42 (20.3) | 35 (17.3) |
| NYHA/WHO FC, n (%) |  |  |  |  |  |
| I | 31 (4.0) | 4 (1.9) | 5 (2.3) | 9 (5.1) | 13 (7.6) |
| II | 189 (24.4) | 39 (18.5) | 56 (25.9) | 45 (25.6) | 49 (28.5) |
| III | 459 (59.2) | 122 (57.8) | 135 (62.5) | 105 (59.7) | 97 (56.4) |
| IV | 96 (12.4) | 46 (21.8) | 20 (9.3) | 17 (9.7) | 13 (7.6) |
| Missing | 125 | 37 | 27 | 31 | 30 |
| WHO Group I diagnosis, n (%) | | | | | |
| APAH – APAH – HIV | 15 (1.7) | 6 (2.4) | 4 (1.6) | 2 (1.0) | 3 (1.5) |
| APAH – Collagen vascular disease/connective tissue disease | 278 (30.9) | 74 (29.8) | 68 (28.0) | 69 (33.3) | 67 (33.2) |
| APAH – Congenital systemic-to-pulmonary shunts | 45 (5.0) | 6 (2.4) | 11 (4.5) | 10 (4.8) | 18 (8.9) |
| APAH – Drugs and toxins | 41 (4.6) | 13 (5.2) | 10 (4.1) | 11 (5.3) | 7 (3.5) |
| APAH – Other | 10 (1.1) | 2 (0.8) | 5 (2.1) | 2 (1.0) | 1 (0.5) |
| APAH – Portal Hypertension | 65 (7.2) | 16 (6.5) | 19 (7.8) | 11 (5.3) | 19 (9.4) |
| FPAH | 22 (2.4) | 3 (1.2) | 8 (3.3) | 4 (1.9) | 7 (3.5) |
| IPAH | 418 (46.4) | 125 (50.4) | 116 (47.7) | 97 (46.9) | 80 (39.6) |
| Pulmonary veno-occlusive disease | 6 (0.7) | 3 (1.2) | 2 (0.8) | 1 (0.5) | 0 |

^a^Patients were deemed newly diagnosed if the qualifying RHC was performed within the 3 months preceding enrollment to REVEAL, and previously diagnosed if the qualifying RHC was prior to the 3 months before enrollment.
APAH: associated PAH; BMI: body mass index; FC: functional class; FPAH: familial PAH; HIV: human immunodeficiency virus; IPAH: idiopathic PAH; IQR: interquartile range; NYHA: New York Heart Association; PAH: pulmonary arterial hypertension; PAPi: pulmonary artery pulsatility index; REVEAL: Registry to Evaluate Early and Long-Term PAH Disease Management; RHC, right heart catheterization; SD: standard deviation; WHO: World Health Organization.
