# Supplementary material for: Predictive Value of the Pulmonary Artery Pulsatility Index in Pulmonary Arterial Hypertension: REVEAL Analysis
Source: Cardiol Res. 2026 Jun 5;17(3):214–26. doi: 10.14740/cr2225 (PMC13278699; doi:10.14740/cr2225)
Supplement: Suppl 5 — Clinical characteristics by PAPi quartile: prevalent group. [file cr-17-03-214-s005.docx]

**Suppl 5.** Clinical Characteristics by PAPi Quartile: Prevalent Group

|  | | **PAPi Quartile** | | | | |
| --- | --- | --- | --- | --- | --- | --- |
| **Characteristic** | **Overall (N = 1,811)** | | **< 3.55 (n = 430)** | **≥ 3.55 to < 5.5 (n = 425)** | **≥ 5.5 to < 9.0 (n = 475)** | **≥ 9.0 (n = 481)** |
| Heart rate at time of vital signs, bpm | | | | | | |
| n | 1,729 | | 412 | 405 | 458 | 454 |
| Mean (SD) | 82.6 (14.2) | | 85.1 (13.8) | 83.3 (14.4) | 81.6 (14.0) | 80.7 (14.3) |
| Median (IQR) | 82.0 (72.0-92.0) | | 84.0 (76.0-93.3) | 83.0 (72.0-93.0) | 81.0 (72.0-91.0) | 80.0 (70.0-90.0) |
| Missing, n | 82 | | 18 | 20 | 17 | 27 |
| Systolic blood pressure, mmHg | | | | | | |
| n | 1,751 | | 415 | 406 | 464 | 466 |
| Mean (SD) | 115.8 (16.9) | | 114.7 (17.3) | 116.2 (17.2) | 116.0 (16.2) | 116.3 (17.0) |
| Median (IQR) | 114.0 (104.0-126.0) | | 112.0 (102.0-125.0) | 114.0 (104.0-125.8) | 112.5 (104.0-126.0) | 116.0 (104.0-126.0) |
| Missing, n | 60 | | 15 | 19 | 11 | 15 |
| Diastolic blood pressure, mmHg | | | | | | |
| n | 1,749 | | 415 | 406 | 464 | 464 |
| Mean (SD) | 69.0 (10.4) | | 69.2 (10.6) | 68.9 (10.9) | 69.2 (9.8) | 69.0 (10.4) |
| Median (IQR) | 69.0 (60.0-76.0) | | 69.0 (61.0-76.0) | 69.0 (60.0-75.0) | 69.0 (62.0-76.0) | 69.0 (60.0-76.0) |
| Missing, n | 62 | | 15 | 19 | 11 | 17 |
| 6-Minute walk distance, m |  | |  |  |  |  |
| n | 1,532 | | 360 | 359 | 408 | 405 |
| Mean (SD) | 379.1 (122.9) | | 359.8 (119.1) | 375.8 (121.1) | 387.3 (129.7) | 391.0 (119.1) |
| Median (IQR) | 387.1 (305.0-456.2) | | 376.6 (293.6-437.3) | 385.0 (299.3-453.5) | 390.1 (306.5-463.0) | 398.0 (327.0-457.2) |
| Missing, n | 279 | | 70 | 66 | 67 | 76 |
| BNP Value, pg/mL |  | |  |  |  |  |
| n | 910 | | 227 | 209 | 244 | 230 |
| Mean (SD) | 259.4 (484.5) | | 271.1 (330.3) | 267.7 (409.5) | 299.8 (718.7) | 197.5 (343.1) |
| Median (IQR) | 101.5 (38.0-282.8) | | 138.0 (50.5-377.5) | 115.0 (37.0-292.0) | 96.0 (36.8-246.3) | 72.0 (31.0-238.0) |
| Missing, n | 901 | | 203 | 216 | 231 | 251 |
| BNP, pg/mL |  | |  |  |  |  |
| n | 164 | | 38 | 50 | 40 | 36 |
| Mean (SD) | 1,930.3 (8,150.2) | | 1,378.6 (4,465.8) | 3,853.9 (13,999.1) | 1,159.9 (2,173.2) | 696.9 (883.3) |
| Median (IQR) | 285.5 (107.8-981.8) | | 373.5 (116.8-991.3) | 278.5 (114.0-1361.0) | 297.5 (104.3-937.3) | 313.5 (106.3-908.0) |
| Missing, n | 1,647 | | 392 | 375 | 435 | 445 |
| PAH Risk score (REVEAL 2.0 Risk Calculator) | | | | | | |
| n | 1,811 | | 430 | 425 | 475 | 481 |
| Mean (SD) | 7.3 (2.3) | | 7.7 (2.4) | 7.3 (2.4) | 7.1 (2.4) | 6.9 (2.1) |
| Median (IQR) | 7.0 (6.0-9.0) | | 8.0 (6.0-9.0) | 7.0 (6.0-9.0) | 7.0 (6.0-9.0) | 7.0 (6.0-8.0) |
| Baseline mPAP (at rest), mmHg | | | | | | |
| n | 1,785 | | 426 | 414 | 470 | 475 |
| Mean (SD) | 49.7 (14.9) | | 47.9 (13.7) | 49.8 (14.6) | 50.3 (14.7) | 50.7 (16.1) |
| Median (IQR) | 49.0 (39.0-58.0) | | 47.0 (39.0-57.0) | 49.0 (41.0-58.0) | 50.0 (40.0-59.0) | 49.0 (38.0-60.0) |
| Missing, n | 26 | | 4 | 11 | 5 | 6 |
| Actual most recent mixed venous O_2_ saturation | | | | | | |
| n | 1,176 | | 266 | 274 | 317 | 319 |
| Mean (SD) | 64.8 (9.2) | | 60.4 (10.4) | 64.6 (7.9) | 66.7 (9.1) | 66.8 (7.8) |
| Median (IQR) | 66.0 (59.0-71.0) | | 62.0 (53.3-68.0) | 65.0 (60.0-70.0) | 67.0 (62.0-72.0) | 68.0 (62.0-72.0) |
| Missing, n | 635 | | 164 | 151 | 158 | 162 |
| Cardiac index variable, L/min/m^2^ | | | | | | |
| n | 1,486 | | 369 | 358 | 386 | 373 |
| Mean (SD) | 2.5 (0.8) | | 2.3 (0.9) | 2.4 (0.8) | 2.6 (0.9) | 2.6 (0.7) |
| Median (IQR) | 2.4 (1.9-2.9) | | 2.1 (1.7-2.7) | 2.3 (1.9-2.9) | 2.5 (2.0-3.0) | 2.5 (2.1-3.0) |
| Missing, n | 325 | | 61 | 67 | 89 | 108 |
| PVR, Wood units |  | |  |  |  |  |
| n | 1,659 | | 413 | 394 | 434 | 418 |
| Mean (SD) | 10.0 (7.2) | | 10.3 (6.3) | 9.9 (6.1) | 9.5 (6.2) | 10.4 (9.7) |
| Median (IQR) | 8.5 (5.5-12.7) | | 9.1 (5.5-13.5) | 8.6 (5.7-12.8) | 8.0 (5.4-12.1) | 8.5 (5.5-11.8) |
| Missing, n | 152 | | 17 | 31 | 41 | 63 |
| Baseline PCWP (at rest), mmHg | | | | | | |
| n | 1,739 | | 416 | 407 | 460 | 456 |
| Mean (SD) | 9.9 (4.2) | | 10.5 (4.2) | 10.7 (4.4) | 9.8 (3.8) | 8.7 (4.1) |
| Median (IQR) | 10.0 (7.0-12.0) | | 10.0 (8.0-13.0) | 10.0 (8.0-13.0) | 10.0 (7.0-12.0) | 8.0 (6.0-11.0) |
| Missing, n | 72 | | 14 | 18 | 15 | 25 |
| Glomerular filtration rate at enrollment, mL/min/1.73 m^2^ | | | | | | |
| n | 1,433 | | 344 | 328 | 375 | 386 |
| Mean (SD) | 74.8 (26.4) | | 73.1 (25.1) | 71.6 (27.0) | 77.9 (25.1) | 76.2 (27.9) |
| Median (IQR) | 73.4 (57.0-92.0) | | 71.3 (54.9-90.0) | 70.3 (53.3-89.7) | 75.5 (60.5-95.0) | 75.6 (56.4-94.7) |
| Missing, n | 378 | | 86 | 97 | 100 | 95 |
| Borg Dyspnea Scale^a^ |  | |  |  |  |  |
| n | 1,376 | | 318 | 331 | 371 | 356 |
| Mean (SD) | 2.9 (1.9) | | 3.0 (2.0) | 3.0 (2.0) | 2.9 (1.9) | 2.8 (1.7) |
| Median (IQR) | 3.0 (1.0-4.0) | | 3.0 (1.0-4.0) | 3.0 (2.0-4.0) | 3.0 (2.0-4.0) | 3.0 (1.0-4.0) |
| Missing, n | 435 | | 112 | 94 | 104 | 125 |
| Medical history of obstructive lung disease, n (%) | | | | | | |
| Yes | 236 (13.4) | | 50 (12.1) | 65 (15.8) | 67 (14.6) | 54 (11.4) |
| No | 1,519 (86.6) | | 362 (87.9) | 347 (84.2) | 392 (85.4) | 418 (88.6) |
| Missing | 56 | | 18 | 13 | 16 | 9 |
| Medical history of reactive airways disease, n (%) | | | | | | |
| Yes | 174 (10) | | 42 (10.2) | 46 (11.2) | 39 (8.6) | 47 (10.0) |
| No | 1,574 (90.0) | | 371 (89.8) | 365 (88.8) | 416 (91.4) | 422 (90.0) |
| Missing | 63 | | 17 | 14 | 20 | 12 |
| Medical history of sleep apnea, n (%) | | | | | | |
| Yes | 358 (20.8) | | 103 (25.7) | 104 (25.6) | 89 (19.8) | 62 (13.3) |
| No | 1,365 (79.2) | | 298 (74.3) | 302 (74.4) | 360 (80.2) | 405 (86.7) |
| Missing | 88 | | 29 | 19 | 26 | 14 |
| History of lung transplant, n (%) | | | | | | |
| Yes | 5 (0.3) | | 1 (0.2) | 2 (0.5) | 2 (0.4) | 0 |
| No | 1,806 (99.7) | | 429 (99.8) | 423 (99.5) | 473 (99.6) | 481 (100) |
| History of atrial septostomy, n (%) | | | | | | |
| Yes | 17 (0.9) | | 1 (0.2) | 2 (0.5) | 5 (1.1) | 9 (1.9) |
| No | 1,794 (99.1) | | 429 (99.8) | 423 (99.5) | 470 (98.9) | 472 (98.1) |
| COPD, n (%) |  | |  |  |  |  |
| Yes | 143 (8.0) | | 31 (7.3) | 36 (8.7) | 41 (8.7) | 35 (7.3) |
| No | 1,649 (92.0) | | 396 (92.7) | 379 (91.3) | 430 (91.3) | 444 (92.7) |
| Missing | 19 | | 3 | 10 | 4 | 2 |
| Pulmonary embolism, n (%) |  | |  |  |  |  |
| Yes | 47 (2.6) | | 13 (3.0) | 12 (2.9) | 13 (2.8) | 9 (1.9) |
| No | 1,745 (97.4) | | 414 (97.0) | 403 (97.1) | 458 (97.2) | 470 (98.1) |
| Missing | 19 | | 3 | 10 | 4 | 2 |
| Congenital heart disease, n (%) | | | | | | |
| Yes | 226 (12.5) | | 19 (4.4) | 37 (8.7) | 67 (14.1) | 103 (21.4) |
| No | 1,585 (87.5) | | 411 (95.6) | 388 (91.3) | 408 (85.9) | 378 (78.6) |
| Any prostacyclin, n (%) |  | |  |  |  |  |
| Yes | 816 (45.7) | | 228 (53.5) | 215 (51.9) | 191 (40.6) | 182 (38.3) |
| No | 969 (54.3) | | 198 (46.5) | 199 (48.1) | 279 (59.4) | 293 (61.7) |
| Missing | 26 | | 4 | 11 | 5 | 6 |
| Any phosphodiesterase-5 inhibitor, n (%) | | | | | | |
| Yes | 918 (51.4) | | 212 (49.8) | 217 (52.4) | 253 (53.8) | 236 (49.7) |
| No | 867 (48.6) | | 214 (50.2) | 197 (47.6) | 217 (46.2) | 239 (50.3) |
| Missing | 26 | | 4 | 11 | 5 | 6 |
| Any endothelin receptor antagonist, n (%) | | | | | | |
| Yes | 937 (52.5) | | 219 (51.4) | 199 (48.1) | 258 (54.9) | 261 (54.9) |
| No | 848 (47.5) | | 207 (48.6) | 215 (51.9) | 212 (45.1) | 214 (45.1) |
| Missing | 26 | | 4 | 11 | 5 | 6 |

^a^Scale ranges from 0, where breathing is causing no difficulty at all, through 10, where breathing difficulty is maximal.

BNP: brain natriuretic peptide; bpm: beats per minute; COPD: chronic obstructive pulmonary disease; IQR: interquartile range; mPAP: mean pulmonary artery pressure; PAH: pulmonary arterial hypertension; PAPi: pulmonary artery pulsatility index; PCWP: pulmonary capillary wedge pressure; PVR: pulmonary vascular resistance; REVEAL: Registry to Evaluate Early and Long-Term PAH Disease Management; SD: standard deviation.
