# Supplementary material for: Predictive Value of the Pulmonary Artery Pulsatility Index in Pulmonary Arterial Hypertension: REVEAL Analysis
Source: Cardiol Res. 2026 Jun 5;17(3):214–26. doi: 10.14740/cr2225 (PMC13278699; doi:10.14740/cr2225)
Supplement: Suppl 6 — Clinical characteristics by PAPi quartile: incident group. [file cr-17-03-214-s006.docx]

**Suppl 6.** Clinical Characteristics by PAPi Quartile: Incident Group

|  | | **PAPi Quartile** | | | | |
| --- | --- | --- | --- | --- | --- | --- |
| **Characteristic** | **Overall** **(N = 900)** | | **< 3.55** **(n = 248)** | **≥ 3.55 to < 5.5** **(n = 243)** | **≥ 5.5 to < 9.0** **(n = 207)** | **≥ 9.0** **(n = 202)** |
| Heart rate at time of vital signs, bpm | | | | | | |
| n | 845 | | 231 | 230 | 195 | 189 |
| Mean (SD) | 84.0 (15.7) | | 87.4 (17.1) | 84.2 (14.7) | 82.8 (15.5) | 80.8 (14.4) |
| Median (IQR) | 83.0 (72.0-95.0) | | 86.0 (75.0-100.0) | 84.0 (72.0-94.0) | 82.0 (71.5-92.0) | 80.0 (71.0-92.0) |
| Missing, n | 55 | | 17 | 13 | 12 | 13 |
| Systolic blood pressure, mmHg | | | | | | |
| n | 842 | | 232 | 230 | 193 | 187 |
| Mean (SD) | 119.6 (18.7) | | 117.0 (19.4) | 120.0 (18.1) | 121.7 (19.2) | 120.3 (17.7) |
| Median (IQR) | 118.0 (106.0-130.8) | | 114.0 (104.0-127.3) | 118.0 (106.0-132.0) | 118.0 (108.0-132.0) | 120.0 (108.0-132.0) |
| Missing, n | 58 | | 16 | 13 | 14 | 15 |
| Diastolic blood pressure, mmHg | | | | | | |
| n | 842 | | 232 | 230 | 193 | 187 |
| Mean (SD) | 72.7 (12.0) | | 73.4 (12.8) | 71.9 (12.0) | 73.2 (12.1) | 72.2 (10.7) |
| Median (IQR) | 72.0 (64.0-80.0) | | 72.0 (64.0-80.3) | 70.0 (64.0-80.0) | 71.0 (64.0-80.0) | 72.0 (66.0-79.0) |
| Missing, n | 58 | | 16 | 13 | 14 | 15 |
| Most recent 6-minute walk distance test, m | | | | | | |
| n | 607 | | 149 | 168 | 145 | 145 |
| Mean (SD) | 317.5 (128.2) | | 292.7 (130.0) | 310.6 (134.7) | 318.9 (123.1) | 349.6 (117.7) |
| Median (IQR) | 327.0 (228.8-411.2) | | 299.0 (209.0-390.0) | 308.0 (217.1-407.3) | 329.0 (231.0-411.5) | 357.0 (278.0-434.0) |
| Missing, n | 293 | | 99 | 75 | 62 | 57 |
| BNP Value, pg/mL |  | |  |  |  |  |
| n | 405 | | 112 | 103 | 94 | 96 |
| Mean (SD) | 467.4 (823.7) | | 745.7 (1233.4) | 442.6 (750.1) | 334.9 (388.7) | 299.1 (466.5) |
| Median (IQR) | 225.0 (83.0-530.0) | | 426.5 (158.3-847.0) | 207.0 (60.5-433.0) | 206.0 (83.0-444.8) | 128.0 (58.3-341.0) |
| Missing, n | 495 | | 136 | 140 | 113 | 106 |
| Most recent BNP, pg/mL |  | |  |  |  |  |
| n | 115 | | 36 | 33 | 24 | 22 |
| Mean (SD) | 2,598.7 (4,258.5) | | 2,661.4 (2,276.0) | 3,993.7 (7,161.0) | 1,752.5 (1,847.8) | 1,326.5 (1,533.9) |
| Median (IQR) | 1,240.0 (542.5-3,358.5) | | 1,717.5 (1,102.0-4,285.5) | 1,498.0 (444.0-3,468.0) | 977.5 (490.5-2,452.8) | 698.5 (287.0-2,130.3) |
| Missing, n | 785 | | 212 | 210 | 183 | 180 |
| PAH Risk score (REVEAL 2.0 Risk Calculator) | | | | | | |
| n | 900 | | 248 | 243 | 207 | 202 |
| Mean (SD) | 8.3 (2.2) | | 9.1 (2.2) | 8.3 (2.0) | 8.1 (2.1) | 7.7 (2.1) |
| Median (IQR) | 8.0 (7.0-10.0) | | 9.0 (8.0-11.0) | 8.0 (7.0-10.0) | 8.0 (6.0-10.0) | 8.0 (6.0-9.0) |
| Most recent baseline mPAP at rest, entered or calculated (range restricted) | | | | | | |
| n | 869 | | 239 | 235 | 200 | 195 |
| Mean (SD) | 49.3 (13.3) | | 50.0 (12.7) | 48.7 (12.6) | 51.1 (13.8) | 47.2 (14.1) |
| Median (IQR) | 48.0 (40.0-57.0) | | 50.0 (41.0-59.0) | 48.0 (39.5-58.0) | 50.0 (40.8-59.0) | 45.0 (38.5-53.0) |
| Missing, n | 31 | | 9 | 8 | 7 | 7 |
| Actual most recent mixed venous O_2_ saturation | | | | | | |
| n | 565 | | 167 | 148 | 125 | 125 |
| Mean (SD) | 61.6 (10.5) | | 55.4 (11.1) | 62.0 (10.4) | 64.0 (8.8) | 67.1 (6.9) |
| Median (IQR) | 62.0 (55.0-68.0) | | 56.0 (48.0-62.5) | 62.0 (54.0-69.0) | 64.0 (59.0-69.0) | 67.0 (63.0-71.0) |
| Missing, n | 335 | | 81 | 95 | 82 | 77 |
| Most recent cardiac index (minimum output – not preferred analysis variable), L/min/m^2^ | | | | | | |
| n | 707 | | 200 | 186 | 156 | 165 |
| Mean (SD) | 2.2 (0.7) | | 1.9 (0.7) | 2.3 (0.8) | 2.2 (0.6) | 2.4 (0.7) |
| Median (IQR) | 2.1 (1.7-2.6) | | 1.8 (1.4-2.3) | 2.2 (1.7-2.6) | 2.2 (1.8-2.6) | 2.4 (2.0-2.8) |
| Missing, n | 193 | | 48 | 57 | 51 | 37 |
| Most recent PVR (Fick hierarchy – preferred analysis variable), Wood units | | | | | | |
| n | 857 | | 239 | 227 | 198 | 193 |
| Mean (SD) | 10.9 (6.3) | | 12.1 (7.2) | 10.0 (5.6) | 11.4 (6.2) | 9.9 (5.4) |
| Median (IQR) | 9.6 (6.3-13.8) | | 10.6 (6.9-16.3) | 8.6 (5.7-13.4) | 10.3 (6.6-14.2) | 8.8 (6.4-11.8) |
| Missing, n | 43 | | 9 | 16 | 9 | 9 |
| Most recent baseline PCWP, at rest (range restricted) | | | | | | |
| n | 846 | | 229 | 226 | 199 | 192 |
| Mean (SD) | 9.6 (3.7) | | 11.0 (3.2) | 10.2 (3.6) | 9.4 (3.6) | 7.2 (3.5) |
| Median (IQR) | 10.0 (7.0-12.0) | | 12.0 (9.0-13.0) | 10.0 (8.0-13.0) | 9.0 (7.0-11.5) | 7.0 (5.0-10.0) |
| Missing, n | 54 | | 19 | 17 | 8 | 10 |
| Glomerular filtration rate at enrollment, mL/min/1.73 m^2^ | | | | | | |
| n | 725 | | 201 | 198 | 166 | 160 |
| Mean (SD) | 75.3 (26.6) | | 74.3 (28.0) | 75.9 (27.7) | 71.8 (24.2) | 79.5 (25.5) |
| Median (IQR) | 74.0 (56.2-96.2) | | 72.9 (53.4-97.7) | 72.8 (56.2-99.1) | 69.9 (54.0-89.0) | 78.6 (60.9-98.9) |
| Missing | 175 | | 47 | 45 | 41 | 42 |
| Borg Dyspnea Scale |  | |  |  |  |  |
| n | 546 | | 134 | 153 | 130 | 129 |
| Mean (SD) | 3.3 (2.1) | | 3.4 (2.1) | 3.8 (2.1) | 3.3 (2.1) | 2.8 (1.8) |
| Median (IQR) | 3.0 (2.0-4.0) | | 3.0 (2.0-5.0) | 4.0 (3.0-5.0) | 3.0 (2.0-5.0) | 3.0 (1.0-4.0) |
| Missing, n | 354 | | 114 | 90 | 77 | 73 |
| Medical history of obstructive lung disease, n (%) | | | | | | |
| Yes | 150 (17.2) | | 40 (16.7) | 40 (16.9) | 40 (19.9) | 30 (15.2) |
| No | 724 (82.8) | | 199 (83.3) | 197 (83.1) | 161 (80.1) | 167 (84.8) |
| Missing | 26 | | 9 | 6 | 6 | 5 |
| Medical history of reactive airways disease, n (%) | | | | | | |
| Yes | 83 (9.7) | | 18 (7.7) | 21 (9.1) | 22 (11.2) | 22 (11.4) |
| No | 772 (90.3) | | 216 (92.3) | 210 (90.9) | 175 (88.8) | 171 (88.6) |
| Missing | 45 | | 14 | 12 | 10 | 9 |
| Medical history of sleep apnea, n (%) | | | | | | |
| Yes | 195 (23.4) | | 66 (28.9) | 63 (27.3) | 37 (19.6) | 29 (15.7) |
| No | 638 (76.6) | | 162 (71.1) | 168 (72.7) | 152 (80.4) | 156 (84.3) |
| Missing | 67 | | 20 | 12 | 18 | 17 |
| History of lung transplant, n (%) | | | | | | |
| Yes | 0 | | 0 | 0 | 0 | 0 |
| No | 900 (100) | | 248 (100) | 243 (100) | 207 (100) | 202 (100) |
| History of atrial septostomy, n (%) | | | | | | |
| Yes | 1 (0.1) | | 0 | 0 | 1 (0.5) | 0 |
| No | 899 (99.9) | | 248 (100) | 243 (100) | 206 (99.5) | 202 (100) |
| COPD, n (%) |  | |  |  |  |  |
| Yes | 123 (14.0) | | 32 (13.2) | 39 (16.5) | 31 (15.3) | 21 (10.6) |
| No | 756 (86.0) | | 210 (86.8) | 198 (83.5) | 171 (84.7) | 177 (89.4) |
| Missing | 21 | | 6 | 6 | 5 | 4 |
| Pulmonary embolism, n (%) |  | |  |  |  |  |
| Yes | 35 (4.0) | | 11 (4.5) | 11 (4.6) | 6 (3.0) | 7 (3.5) |
| No | 844 (96.0) | | 231 (95.5) | 226 (95.4) | 196 (97.0) | 191 (96.5) |
| Missing | 21 | | 6 | 6 | 5 | 4 |
| Congenital heart disease, n (%) | | | | | | |
| Yes | 45 (5.0) | | 6 (2.4) | 11 (4.5) | 10 (4.8) | 18 (8.9) |
| No | 855 (95.0) | | 242 (97.6) | 232 (95.5) | 197 (95.2) | 184 (91.1) |
| Any prostacyclin, n (%) |  | |  |  |  |  |
| Yes | 197 (22.6) | | 82 (34.0) | 50 (21.3) | 39 (19.5) | 26 (13.2) |
| No | 676 (77.4) | | 159 (66.0) | 185 (78.7) | 161 (80.5) | 171 (86.8) |
| Missing | 27 | | 7 | 8 | 7 | 5 |
| Any phosphodiesterase-5 inhibitor, n (%) | | | | | | |
| Yes | 321 (36.8) | | 88 (36.5) | 77 (32.8) | 75 (37.5) | 81 (41.1) |
| No | 552 (63.2) | | 153 (63.5) | 158 (67.2) | 125 (62.5) | 116 (58.9) |
| Missing | 27 | | 7 | 8 | 7 | 5 |
| Any endothelin receptor antagonist, n (%) | | | | | | |
| Yes | 181 (20.7) | | 40 (16.6) | 42 (17.9) | 49 (24.5) | 50 (25.4) |
| No | 692 (79.3) | | 201 (83.4) | 193 (82.1) | 151 (75.5) | 147 (74.6) |
| Missing | 27 | | 7 | 8 | 7 | 5 |

BNP: brain natriuretic peptide; bpm: beats per minute; COPD: chronic obstructive pulmonary disease; IQR: interquartile range; mPAP: mean pulmonary artery pressure; PAH: pulmonary arterial hypertension; PAPi: pulmonary artery pulsatility index; PCWP: pulmonary capillary wedge pressure; PVR: pulmonary vascular resistance; REVEAL: Registry to Evaluate Early and Long-Term PAH Disease Management; SD: standard deviation.
