# Supplementary material for: Predictive Value of the Pulmonary Artery Pulsatility Index in Pulmonary Arterial Hypertension: REVEAL Analysis
Source: Cardiol Res. 2026 Jun 5;17(3):214–26. doi: 10.14740/cr2225 (PMC13278699; doi:10.14740/cr2225)
Supplement: Suppl 8 — Mortality according to PAPi quartile (incident and prevalent patients). [file cr-17-03-214-s008.docx]

**Suppl 8.** Mortality according to PAPi quartile

**
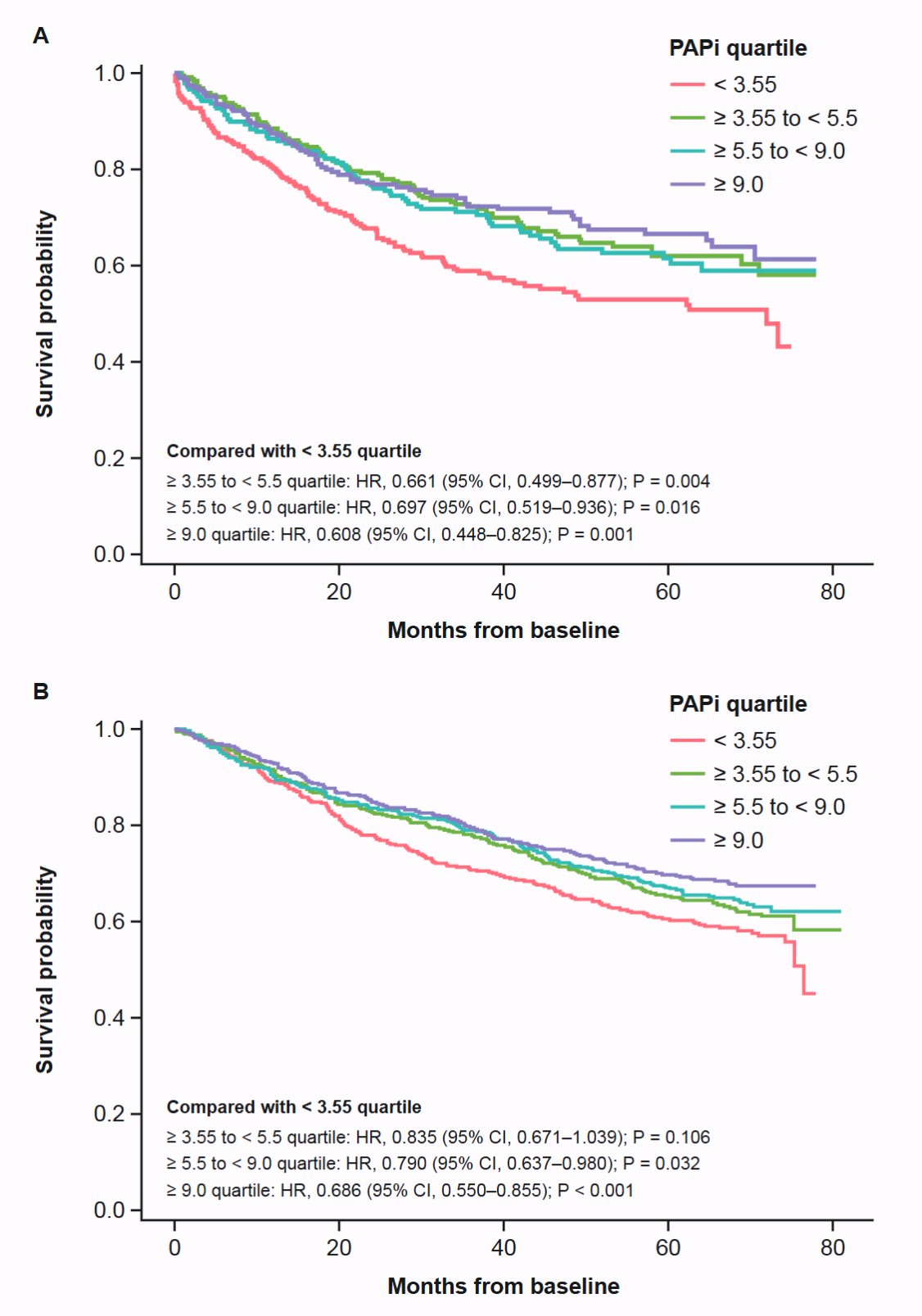
**

(A) Incident and (B) prevalent patients.

Incident cases were defined as patients who received a diagnosis of PAH confirmed by RHC during study recruitment; prevalent cases were defined as patients diagnosed prior to the start of the study [1].

CI: confidence interval; HR: hazard ratio; PAH, pulmonary arterial hypertension; PAPi: pulmonary artery pulsatility index; RHC: right heart catheterization.

**Reference**

1. McGoon MD, Miller DP. REVEAL: a contemporary US pulmonary arterial hypertension registry. Eur Respir Rev. 2012;21(123):8-18.
