# Supplementary figures and images for: Predictive Value of the Pulmonary Artery Pulsatility Index in Pulmonary Arterial Hypertension: REVEAL Analysis
Source: Cardiol Res. 2026 Jun 5;17(3):214–26. doi: 10.14740/cr2225 (PMC13278699; doi:10.14740/cr2225)

**Suppl 9.** Histogram of PAPi scores/values


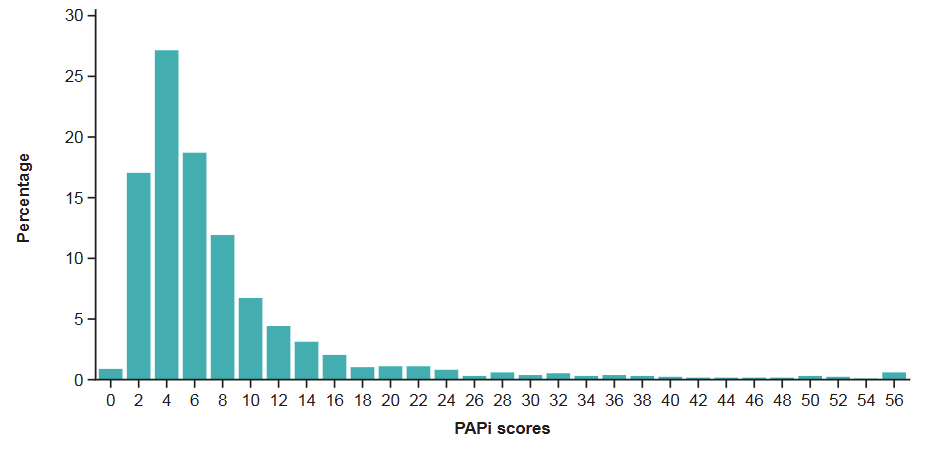


PAPi: pulmonary artery pulsatility index.

Supplement: Suppl 9 — Histogram of PAPi scores/values. [file cr-17-03-214-s009.docx]
